# Exploring the Impact of Fermentation Time and Climate on Quality of Cocoa Bean-Derived Chocolate: Sensorial Profile and Volatilome Analysis

**DOI:** 10.3390/foods13162614

**Published:** 2024-08-20

**Authors:** Sandra Llano, Fabrice Vaillant, Margareth Santander, Andrés Zorro-González, Carlos E. González-Orozco, Isabelle Maraval, Renaud Boulanger, Sebastián Escobar

**Affiliations:** 1 Corporación Colombiana de Investigación Agropecuaria (Agrosavia), Process & Quality Cocoa Laboratory, Centros de Investigación La Selva, Palmira and La Libertad—Km 14 Mosquera-Bogotá, Mosquera 250047, Colombia; llanogils@gmail.com (S.L.); fabrice.vaillant@cirad.fr (F.V.); msantander@agrosavia.co (M.S.); azorro@agrosavia.co (A.Z.-G.); cegonzalez@agrosavia.co (C.E.G.-O.); 2 Centre de Coopération Internationale en Recherche Agronomique pour le Développement—CIRAD, UMR QualiSud, 1, F-34398 Montpellier, France; isabelle.maraval@cirad.fr (I.M.); renaud.boulanger@cirad.fr (R.B.); 3 UMR Qualisud, Univ Montpellier, CIRAD, Université d’Avignon, Université de la Réunion, Montpellier SupAgro, F-34000 Montpellier, France

**Keywords:** fine-flavor chocolate, sensory quality, volatilome, fermentation time and agroclimatic relationship with quality

## Abstract

The market for fine-flavor cocoa provides significant benefits to farmers. However, identifying the sensory qualities of chocolate under specific environmental conditions and measuring how its chemical compounds may be affected by climate differences and postharvesting practices remain a challenge. This study investigates how fermentation time and agroclimatic conditions in Colombia’s fine cocoa-producing region of Arauca influence the sensory profile and volatile compound composition (volatilome) of chocolate derived from cocoa beans. Sensory evaluation was conducted on chocolates fermented for 48, 72, 96, and 120 h, revealing that fermentation time critically affects the development of fine-flavor attributes, particularly fruitiness and nuttiness. The optimal fermentation period to enhance these attributes was identified at 96 h, a duration consistently associated with peak fruitiness under all studied climatic conditions. Analysis of 44 volatile compounds identified several key aroma markers, such as acetoin, 1-methoxy-2-propyl acetate, and various pyrazines, which correlate with desirable sensory attributes. These compounds exhibited varying amounts depending on fermentation time and specific agroclimatic conditions, with a 96 h fermentation yielding chocolates with a higher quantity of volatile compounds associated with preferred attributes. Our findings highlight the complex interaction between fermentation processes and agroclimatic factors in determining cocoa quality, providing new insights into optimizing the flavor profiles of chocolate.

## 1. Introduction

Chocolate, a highly esteemed product globally, experiences diverse market segmentation, primarily between bulk and fine cocoa. The latter is prized for its unique aromas, including fruitiness, florality, herbal, wood, nuttiness, or caramel notes, offering a rich and balanced chocolate experience [1,2]. These distinctive flavor profiles, governed by volatile and nonvolatile fractions, meet the consumer’s growing demand for fine-flavor chocolates [3,4,5]. In this context, Colombia emerges as a significant player in the realm of fine and flavored cocoa production. Cocoa cultivation in Colombia not only represents a promising avenue for economic development but also serves as a transformative strategy for replacing illicit crops, earning it the moniker “the crop for peace” [6]. The country’s potential for commercializing fine and flavored cocoa is substantial. The International Cocoa Organization acknowledges that up to 95% of Colombian cocoa destined for export has the potential to be marketed as fine or flavored cocoa. Leveraging this potential necessitates the integration of Colombia’s unique origin advantages with standardized and well-managed postharvest cocoa operations, aiming to enhance the quality of the final product. This strategic approach aligns with the global consumer demand for chocolates with distinct and refined flavor profiles, positioning Colombia to capitalize on its capability to produce cocoa that meets these high standards. To make the most of this potential, it is essential to integrate the country’s origin advantages with postharvest cocoa operations to maximize the quality. Cocoa quality is influenced by different factors, such as agroclimatic conditions, genetic diversity [7,8], and postharvest practices [9,10,11,12,13]. Several studies have mentioned that agroclimatic conditions and geographical origin exert influence on the morphoagronomic development of cocoa fruits, but the direct relationship between agroclimatic conditions and sensory and volatile profiles has not been conclusively established.

Fermentation is a critical stage in cocoa processing, transforming endogenous seed components into flavor precursor metabolites. These metabolites are key to forming the volatile compounds that define chocolate’s aroma and sensory attributes during roasting [12,14,15]. Volatile aroma compounds, including esters, alcohols, acids, phenols, pyrazines, aldehydes, and ketones, contribute to the complex flavor profile of cocoa products, offering a range of sweet, sour, fruity, floral, nutty, earthy, and roasted notes [16,17]. In this sense, fermentation conditions influence the formation of flavor precursor compounds and, consequently, their quality. Monitoring volatile compounds and precursor metabolites over time offers valuable insights into the development and evolution of flavor during fermentation [3,18,19,20,21].

No comprehensive characterization has been conducted to determine or classify cocoa quality based on cocoa variety, regional or local climate, combined with sensory indicators, and the analysis of volatile compounds over fermentation time.

The present study focuses on exploring climatic variations in 1800 cacao farms in Arauca, Colombia, to establish production clusters, looking for the optimal fine-flavor product through the positive impact of climatic conditions and fermentation on flavor formation [10,13,20,22,23] by analyzing variations in the sensory profile and the identification and quantification of volatile aromatic compounds. This study highlights the identification of discriminating aroma compounds in chocolates produced from cocoa beans subjected to different fermentation times and grown under different climatic conditions. This study, grounded in the context of Colombia’s diverse cacao-producing regions, offers a methodological framework that can be adapted and applied by cacao-origin countries worldwide, aiming to explore similar correlations between climatic conditions, fermentation processes, and cocoa quality. Furthermore, this study promises significant benefits for cacao farmers by identifying optimal conditions for fine-flavor cocoa production, thereby enhancing the quality and market appeal of their products. Importantly, the findings also lay a foundational basis for cacao producers to support or establish a potential designation as a denomination of origin for cacao [23], leveraging unique regional characteristics to further differentiate their products in the global market.

## 2. Materials and Methods

### 2.1. Agroclimatic Classification

The Department of Arauca is the second-largest cocoa-producing region in Colombia, with great importance in the production of fine cocoa in the country. The generation of climatic maps allows for an increase in the knowledge and technology available in the region, integrating data on the presence of cocoa farms with climatic variables, which becomes a powerful tool. This is why we rely on a previous study by Gonzalez-Orozco et al. [24], which aimed at providing a database of the spatial distribution of cocoa farms in Arauca and agroclimatic maps that identify and locate the climatic regions of cocoa in Arauca.

Seven climate [25] variables were used to conduct the statistical analysis of such variables against the predetermined cacao regional clusters in Arauca [24]: wind speed, relative humidity, minimum temperature, maximum temperature, average temperature, annual precipitation, and solar radiation. Furthermore, 11 soil temperature [26] variables (SBIO1_mean annual temperature, SBIO2_average diurnal range, SBIO3_isothermality, SBIO4_temperature seasonality, SBIO5_maximum temperature of the hottest month, SBIO6_minimum temperature of the coldest month, SBIO7_annual temperature range, SBIO8_mean temperature of the wettest month, SBIO9_mean temperature of the driest monthly quarter, SBIO10_mean temperature of the wettest monthly quarter, SBIO11_mean temperature of the coldest monthly quarter) were used at a depth of 0–5 cm. The spatial resolution of the environmental variables was 1 km^2^.

#### Statistical Analysis

A descriptive and exploratory analysis was performed with all 7 climate and 11 soil temperature variables for the 1800 cacao farms under study. The analysis included central tendency measurements, data dispersion, and normal distribution verification. A Kruskal–Wallis analysis was performed using all climate and soil temperature variables. To identify statistical differences between climate clusters, a one-way analysis of variance (ANOVA) and Tukey’s HSD test comparisons were conducted between the categorical climate clusters previously identified and the climate and soil temperature variables. The computed parameters were performed with α = 0.05. The result of classifying 1800 farms allowed for the identification of nine agroclimatic cacao production clusters in Arauca using the 7 climate variables and the 11 soil temperature variables [24].

### 2.2. Fermentation of Cocoa Seeds

Due to logistical reasons, it was not possible to use the full 1800 farms of the nine agroclimatic regions in this study. Consequently, a selection of 180 representative farms, 20 farms from each of the nine regions, was selected. This approach enabled us to conduct a more robust and consistent harvest campaign across the regions. The research aimed to investigate the potential impact of agroclimatic variability on the sensory profile and volatilome of chocolate derived from cocoa beans. Harvesting involved selecting a representative sample of cacao cultivars homogeneously spread from each farm, focusing on various local hybrids and renowned varieties from Arauca known for winning global competitions, such as FSA13, FEAR5, FTA2, and commercial clones, such as ICS1, ICS95, and TSH565. Therefore, it is assumed that if differences were observed, they could not be attributed to genetic factors. Doing this allows us to explore the potential effects of climate-related variables on the quality of the chocolate obtained from the nine cocoa production zones or clusters. Only healthy and fully ripe cocoa pods were harvested one day before the fermentation process and then transported to the central postharvest processing site. The fermentation process was developed as reported previously in standardized wooden box [10,12,23], with some modifications. Cocoa pods were carefully opened, and the seeds were manually extracted and mixed to form an initial batch containing all the collected cultivars. From this batch, 150 kg was allocated into three separate 50 kg batches. Each batch of cocoa seeds was placed in independent compartments of a previously validated wooden fermenter system [10,12]. Therefore, in each region, the three fermentation batches served as replicates, representing a mix of cocoa seeds from different farms. The first aeration of the cocoa mass occurred manually after 72 h using a wooden shovel, followed by subsequent mixing every 12 h until reaching 120 h of fermentation across all nine fermentations.

#### Cocoa Sampling and Drying

A sampling of 1500 g of cocoa seeds was made from the center of the mass for each fermentation process at 48, 72, 96, and 120 h. All samples were stored at −20 °C. Samples were subsequently dried by forced convection with hot air at 50 °C for 72 h in an oven (Memmert, DO6836, (Schwabach, Germany) to produce chocolate and perform sensory and volatile analyses.

### 2.3. Cocoa Sensory Analysis

Chocolates were produced under a standard protocol previously described by Santander et al. [12] with some modifications at the Center de Coopération Internationale en Recherche Agronomique pour le Développement (CIRAD) chocolate factory laboratory (Montpellier, France). Cocoa beans were roasted at 125 °C for 25 min in an oven (Memmert, UL 30, Schwabach, Germany). The roasted cocoa nibs were subsequently cracked in limprimita cocoa breaker (Capco Cocoabreaker240-1, Ipswich, UK) and deshelled using a cocoa winnower machine (Capco Cocoawinnow, Ipswich, UK). The cocoa nibs were grinded by using a mortar and pestle alumina grinder (Capco Mortarmill1SS, Ipswich, UK) over 60 min. Sugar was added after 30 min of grinding. A three-roll refiner (Exakt 80S, City, Germany) for refining stage was used. The refining stage was performed twice. The refined chocolate mass was conched for 2 h at 70 °C with 5% cocoa butter and 1% soy lecithin in a longitudinal conching machine with a rotating roller (ZumWald LR01, Erlenbach, Switzerland). The tempering stage comprised heating the chocolate above 42 °C, cooling it to 32 °C, reaching 29 °C, increasing to 31 °C, and finally heating it to 32 °C before being molded.

A descriptive sensory analysis was performed using the consensus profile method (International Organization for Standardization, 2016) in the sensory analysis laboratory at CIRAD. The chocolate samples were blindly tasted by nine trained judges with experience evaluating chocolate.

There were two types of evaluation sessions. First, the judges agreed on a vocabulary with sensory attributes to evaluate the samples, and 11 descriptors were selected to describe the flavor of the chocolate samples. The descriptors were classified as basic flavors (sweetness, acidity, bitterness, and astringency; fine flavors: cocoa, fruitiness, nuttiness, floral, and spices) and off-flavors (burned and animal). Finally, global quality was also considered. This parameter represents the overall impression of the chocolate, encompassing all sensory elements, including the presence and significance of atypical aromas and special characteristics. In the second session, the judges were seated in individual booths, and the chocolates (5 g) were served at 20 °C ± 1 °C on identical plates prelabeled with a random three-digit code. Samples were presented successively, one by one, in a monadic presentation order. A completely randomized design was used for the sample presentation. Judges scored chocolate samples using a 10-point scale from 0 (not perceivable) to 10 (very intense) regarding the sensory attributes defined initially. For neutralization between samples, unsalted crackers and noncarbonated water were available.

### 2.4. Volatile Compound Analysis in Chocolate

The volatile organic compounds (VOCs) of chocolate samples (2.0 g) were extracted using solid-phase microextraction in the headspace (SPME-HS), as described by Assi-Clair et al. [27] using a 50/30 µm divinylbenzene/carboxene/polydimethylsiloxane (DVB/CAR/PDMS) fiber provided by Supelco. The fiber was preconditioned in the chromatograph injector at 250 °C for 3 min, and then, to reach equilibrium, it was exposed to the chocolate sample headspace at 50 °C for 45 min.

The volatile compounds were analyzed using an Agilent 6890 N gas chromatography mass spectrometry (GC-MS) instrument equipped with a Hewlett Packard capillary column DBWAX, 60 m length × 0.25 mm internal diameter × 0.25 µm film thickness (Agilent, Palo Alto, CA, USA). The GC oven temperature was initially set at 40 °C for 5 min, increased to 140 °C at a rate of 2 °C/min, and then increased at a rate of 10 °C/min to 250 °C for 66 min. The carrier gas was high-purity helium at 1 mL min^−1^. The splitless injection mode was at 250 °C (2 min). The selective mass detector was a quadrupole (Hewlett Packard, Model 5973) with an electronic impact ionization system at 70 eV and 230 °C [27]. VOCs were tentatively identified using the two criteria previously defined: (i) comparison of the retention index with the CIRAD aromatic database; (ii) probability-based matching of mass spectra with those obtained from the NIST17 library. For each chocolate sample, three replicates were conducted. The relative amount of each compound was calculated based on the exact weight of the sample and the known concentration of the butanol added as an internal standard. Then, 100 µL of a 600 mg/L butanol solution was added. Each sample was analyzed in triplicate, and the mean of the three obtained ratios per sample was reported for subsequent statistical analysis. The relative amount of each compound was expressed as the ratio of the compound’s area to the area of the butanol (internal standard).

### 2.5. Data Analysis

Multivariate analyses were made using SIMCA 13.0.2.0 software (Umetrics, Umeå, Sweden). Afterward, a supervised partial least squares discriminant analysis (OSC-PLS-DA) was applied using fermentation time groups (48, 72, 96, and 120 h) to obtain discriminating VOC quality markers. The discriminant power of each VOC was evaluated by the variable importance in projection (VIP) score. The first 15 VOCs with VIP > 1.0 were considered relevant.

The significance of this model was assessed using a cross-validated analysis of variance *p*-value (CV-ANOVA), and its quality was evaluated via the parameters R2X, R2Y, and Q2X. A permutation test (*n* = 100) was conducted to evaluate data overfitting. A variable importance projection index (VIP index) was assigned to each variable (VOC) to show the discrimination power between the different groups of the PLS-DA model. Overall, VOCs that were selected to be studied displayed a VIP value higher than 1 and an ANOVA *p*-value lower than 0.05.

## 3. Results and Discussion

### 3.1. Climatic Analysis

The Kruskal–Wallis H test revealed a significant difference in the dependent variables of climate and soil temperature among the various groups (χ2(7) = 1004.68, *p* < 0.001; χ2(11) = 1456.74, *p* < 0.001).

After analyzing the set of climate variables, only precipitation, wind speed, and maximum temperature exhibited statistical significance following the ANOVA test among the seven climate variables. Regarding soil temperature, all variables displayed significant differences among the nine climate cacao production clusters. However, it is not anticipated that all comparisons between clusters, climate, and soil temperature variables would yield significant differences.

**Figure 1:**
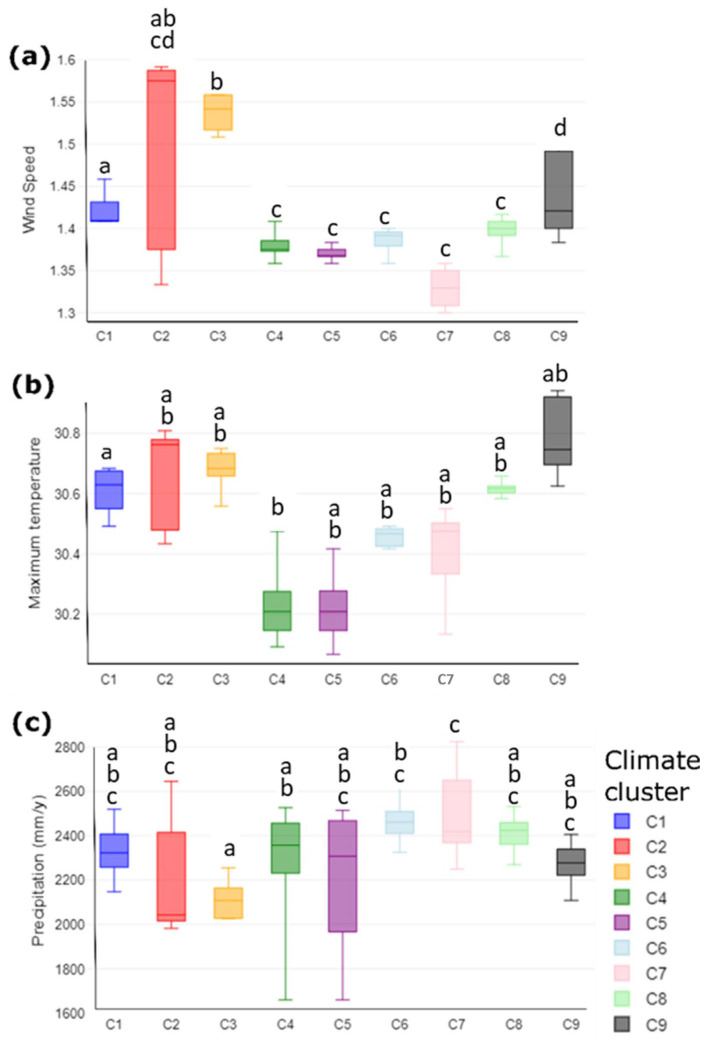
Multiple comparisons in climate variables in the 180 farms classified as nine clusters in Arauca. Values with the same letter are not statistical differences among clusters (ANOVA, Tukey’s test, *p* < 0.05). Charts framed in a rectangle mean variables statistically significant. (**a**) *p*-value = 0.0439869, [*p*(x ≤ F) = 0.956013]; (**b**) *p*-value = 0.0462364, [*p*(x ≤ F) = 0.953764]; (**c**) *p*-value = 0.0000992048, [*p*(x ≤ F) = 0.999901].

**Figure 2:**
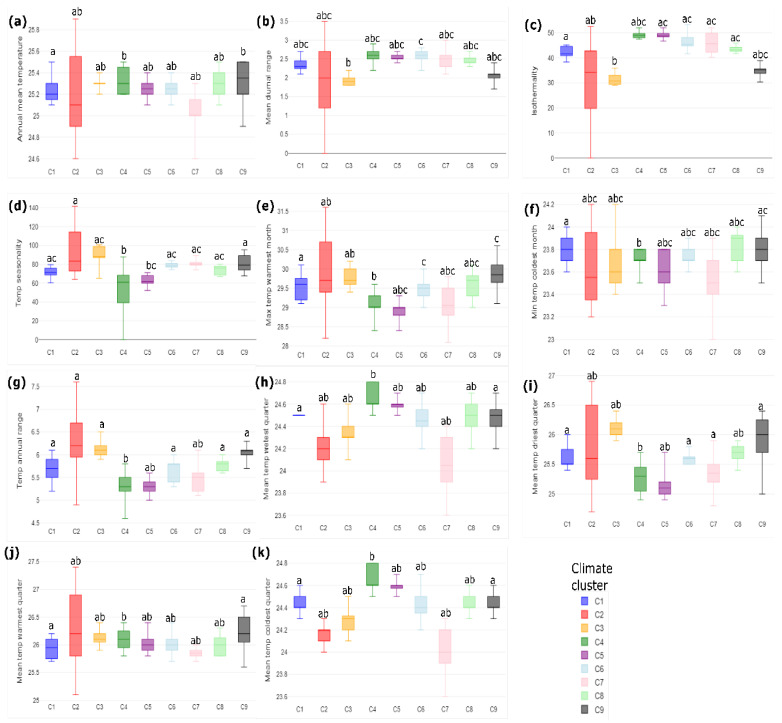
Multiple comparisons in soil temperature in the 180 farms classified as nine clusters in Arauca. Values with the same letter are not statistical differences among clusters (ANOVA, Tukey’s test, *p* < 0.05). (**a**) SBIO1_Annual mean temperature, *p*-value = 0.0232847, [*p*(x ≤ F) = 0.976715 ]; (**b**) SBIO2_Mean diurnal range, *p*-value = 0.00147094, [*p*(x ≤ F) = 0.998529]; (**c**) SBIO3_Isotermality, *p*-value = 1.44085 × 10^−7^, [*p*(x ≤ F) = 1]; (**d**) SBIO4_Temperature seasonality, *p*-value = 0.00000175783, [*p*(x ≤ F) = 0.999998]; (**e**) SBIO5_Max_temperature of warmest month, *p*-value = 0.0108403, [*p*(x ≤ F) = 0.98916]; (**f**) SBIO6_Min_temperature of coldest month, *p*-value= 0.0159335, [*p*(x ≤ F) = 0.984066]; (**g**) SBIO7_Temperature annual range, *p*-value = 0.000327396, [*p*(x ≤ F) = 0.999673]; (**h**) SBIO8_Mean_temperature of wettest quarter, *p*-value = 0.028066, [*p*(x ≤ F) = 0.971934]; (**i**) SBIO9_Mean temperature of driest quarter, *p*-value = 0.0109722, [*p*(x ≤ F) = 0.989028]; (**j**) SBIO10_Mean temperature of warmest quarter, *p*-value = 0.0247223, [*p*(x ≤ F) = 0.975278]; (**k**) SBIO11_Mean temperature of coldest quarter, *p*-value = 0.0319085, [*p*(x ≤ F) = 0.968092].

We identified that the nine predetermined climate clusters for cacao in Arauca (24) have specific climate and soil temperature conditions (Figure 1 and Figure 2). These new results reaffirm that there are significant climate differences among cacao regions in Arauca, allowing us to support the presence of nine agroclimatic clusters of cacao production.

In general, our findings indicate that climate variables play a less influential role than soil temperature variables in potentially explaining the climate variations among the nine predetermined climate clusters in Arauca. Figure 1 illustrates the climatic variables that significantly influence the clustering of the cacao farms in the nine agroclimatic clusters of cacao production.

Focusing on the three significant climate variables, we observe that the area encompassing clusters 2 and 3 in the northern region of Arauca exhibited the highest wind speeds in the entire region, whereas closer to the cordillera, the wind speed was lower (Figure 1a). Clusters 9, 1, and 4 showed significant differences in maximum temperatures for being the hottest of all areas in the region (Figure 1b). Regarding rainfall, clusters 6 and 7 closer to the foothills showed the highest precipitation values, distinctively unlike clusters 2 and 3 in the north of Arauca, which are exposed to less rainfall (Figure 1c).

The influence of soil temperature variables in supporting the presence of the nine clusters was found to be stronger than that of climate variables. Surprisingly, all 11 soil temperature variables exhibited significant differences between them and the nine clusters (Figure 2). This finding suggests that changes in the microclimate of the soil may exert a stronger influence on the cacao genotypes than mere macroclimate variables. Specifically, soil climate variables related to changes in seasonality, as well as maximum and minimum ranges such as isothermality (Figure 2c), temperature seasonality (Figure 2d), and temperature annual range (Figure 2g), demonstrated the strongest influence in explaining the differences among clusters in Arauca.

Remarkably, a substantially similar overarching pattern was observed in the soil temperature analysis, where the cooler and more humid clusters in the western foothills of the cordillera stood distinct from the hotter and drier areas in the eastern zones of Arauquita and Tame. We also noticed a distinct differentiation between the central region, considered a transitional climate zone, and the eastern and western clusters. A differentiation among clusters 9 and 4 is also evident, supporting a north–south pattern of contrasting climates.

### 3.2. Impact of Fermentation Time on the Sensory Profile of Dark Chocolate Samples

**Figure 3:**
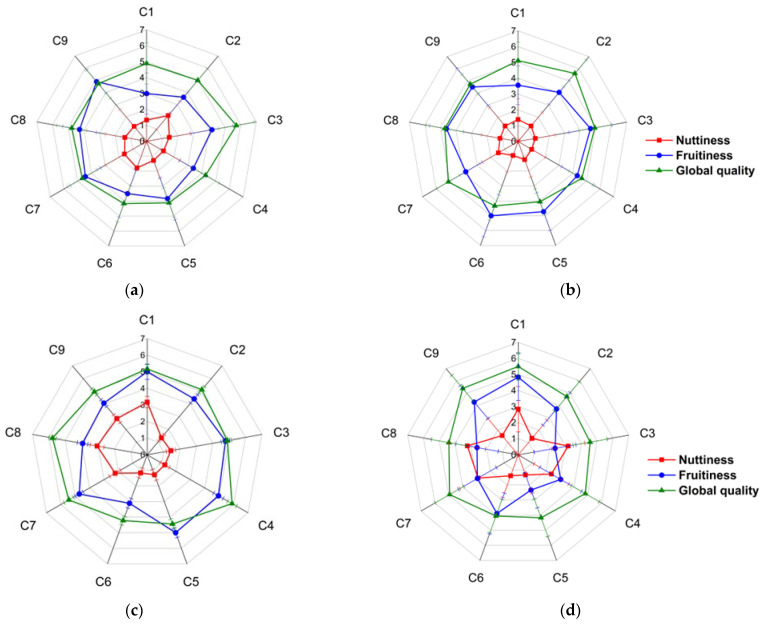
Nuttiness, fruitiness attributes and global quality of chocolates made by fermented cocoa at 48 (**a**), 72 (**b**), 96 (**c**) and 120 h (**d**) of mixtures of cocoa materials from nine cluster (C1, C2, C3, C4, C5, C6, C7, C8 and C9). The numbers correspond to the scoring of the sensory attributes on a 10-point scale 0 (not perceptible) to 10 (very intense).

The sensory attributes analyzed included acidity, bitterness, sweetness, astringency, cocoa, floral fruitiness, nuttiness, animal, and global quality, as evaluated by sensory analysis judges. Detailed results are provided in Supplementary Materials Figures S1 and S2. A key finding is that the sensory profiles of these chocolates exhibited the hallmark features of fine aroma products, particularly those distinguished by their fruity and nutty characteristics. Notably, nuttiness and fruitiness exhibited significant variability across the nine agroclimatic clusters of cocoa production. Global quality scores appear less affected by changes in nuttiness and fruitiness, suggesting that overall satisfaction with the chocolate does not correlate directly with the intensity of these two attributes. Figure 3 shows the variations in fruitiness, nuttiness, and global quality across different agroclimatic clusters at four times along the fermentation process (48, 72, 96 and 120 h).

The nuttiness score increased post-96 h of fermentation in agroclimatic clusters C1, C8, and C9 and showed a rise at 120 h in clusters C3, C4, and C7 while remaining stable in clusters C2, C5, and C6. In contrast, fruitiness was relatively constant across all clusters from 48 to 96 h but diminished significantly at 120 h, with the exception of clusters C1 and C6, which showed no change. Clusters C1, C8, and C9, characterized by higher maximum temperatures (Figure 1b) and average wind speeds (Figure 1a), share similar evapotranspiration conditions, which may influence the nutty flavor profile, as evapotranspiration impacts them [28].

Moreover, the observed sensory predominance of nuttiness in clusters C1, C8, and C9 may be attributable not only to climatic variations but also to geological, soil, and landscape differences. Comparative mapping revealed that clusters C1, C8, and C9 are situated in regions with floodplain terraces and alluvial deposits known for their dry-hot climate and strong winds, unlike cluster 4, which features humid and rainy conditions typical of floodplains near foothills. Despite these observations, no clear pattern emerged regarding the influence of climatic conditions on cocoa bean quality, affirming that cocoa from all nine agroclimatic clusters possesses fine chocolate attributes, such as fruity and nutty notes, with variances stemming from the fermentation process.

Previous research has suggested that location and environmental conditions, including climate, influence flavor development in cacao [7]; however, this has been mentioned, but data evidencing this have not been reported [29,30,31]. This study is the first to explore the relationship between different agroclimatic conditions of cocoa crops for nine climatic production clusters and the flavor quality of the chocolate obtained from them, revealing that fermentation times between 96 and 120 h are optimal for achieving the most intense fine attributes in chocolate flavor.

A fermentation duration of 96 h is identified as the optimal period for maximizing the sensory attributes associated with fine-quality chocolate, despite the persistently high levels of astringency observed at this fermentation time. Although the astringency remains pronounced at 96 h, this timeframe ensures the highest enhancement of desirable characteristics without adversely impacting the overall sensory profile. Beyond 96 h, specifically 120 h, the nuttiness attribute either stabilizes or increases in certain agroclimatic clusters, whereas the fruitiness attribute consistently diminishes across all nine production clusters.

These findings underscore the distinct and inherent qualities of the nine production clusters, which collectively facilitate the production of high-quality cocoa. Notably, chocolates that develop under a 96 h fermentation regime exhibit a pronounced fruity and nutty flavor profile, hallmarking their superior quality. This optimal fermentation time underscores the intricate balance between enhancing desired sensory attributes and mitigating fewer desirable ones, such as astringency, thereby contributing to a nuanced understanding of fermentation’s impact on chocolate quality.

### 3.3. Identification and Quantification of Volatile Aroma Compounds Present in the Dark Chocolate Samples through Fermentation Time

A total of 44 VOCs were identified and quantified for this study, including acids, esters, alcohols, ketones, aldehydes, pyrazines, terpenes, lactones, pyrroles, and furans, similar to those previously reported in other studies [16,21,32,33,34]. The volatile compounds were classified into fruity, floral, nutty/chocolate, buttery/creamy, undesirable, and other volatiles according to their general odor characteristics based on the literature, as shown in Table 1. The relative amounts in chocolate samples are reported in Table S1.

**Figure 4:**
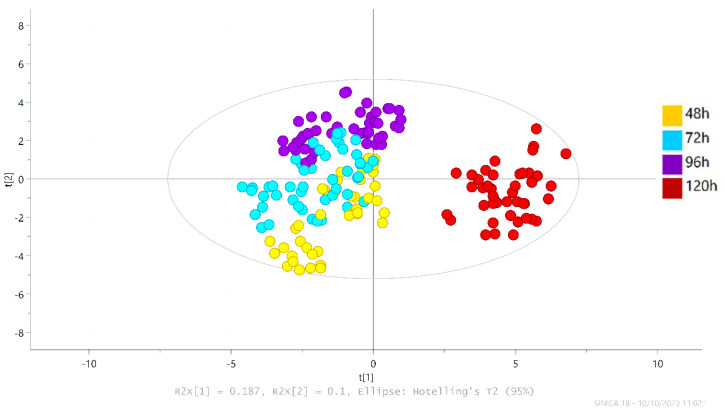
Partial least square discriminant analysis (OSC-PLS-DA) score from volatile profile data of the chocolate samples produced from 48 (yellow points), 72 (blue points), 96 (purple points), and 120 h (red points) fermented beans from nine regions of Arauca. Permutation test for the sample dataset [n = 100; R2 = (0.0, 0.133), Q2 = (0.0, −0.715)].

We used a PLS-DA model that allowed for the discrimination of volatile compounds between fermentation times. An OSC-PLS-DA model was built with different fermentation times, including the nine climatic clusters. Figure 4 shows the model. The model was statistically significant; all validity tests were deemed statistically acceptable (R2Y = 0.717, Q2Y = 0.52). The ANOVA of the cross-validation predictive residual (CV-ANOVA) arose with a low *p*-value (0), and for the permutation test n = 100, it gave a low probability of model overfitting. The result is shown in Figure S3.

The model demonstrated separation based on fermentation time, and we observed changes in the volatilome throughout the fermentation process from 48 to 120 h, with a more marked change between 96 and 120 h, as shown in Figure 4.

The highest discriminant compounds with a VIP ≥ 1 and *p* < 0.05 were 2-pentanol, acetoin, 2,3,5,6-tetramethylpyrazine, benzyl alcohol, 2,3,5-trimethylpirazine, ethyl acetate, 1-mwthoxy-2-propyl acetate, limonene, acetic acid, acetaldehyde, 3,5-dimethyl-2-ethylpirazine, 3-methylbutanoic acid, 2,6-dimethylpyrazine, 2-phenethylpirazine and 2,3-butanediol (Isomer A and B). The VIP values are shown in Table S2 in the Supplementary Material.

**Figure 5:**
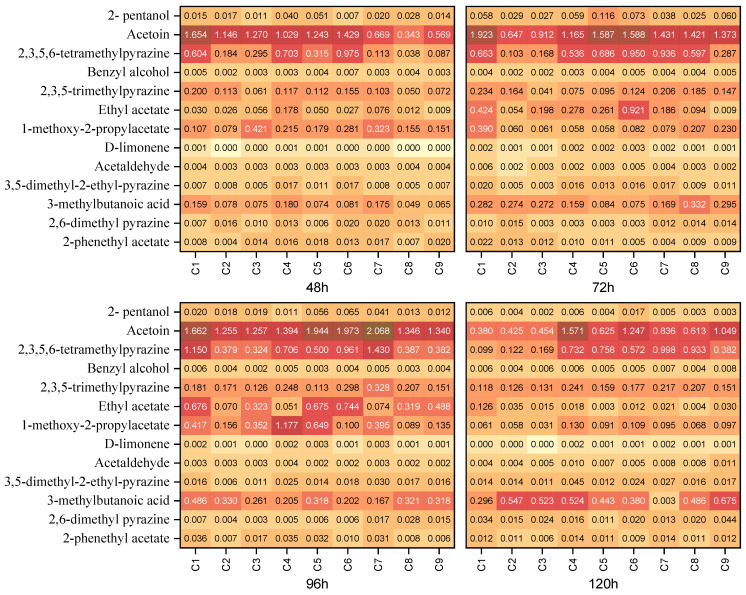
Heatmap of mean relative amount of volatile compounds (ratio of the compound’s area to the area of the butanol) with VIP ≥ 1 (rows) of chocolate samples produced from beans fermented for 48, 72, 96, and 120 h from nine regions of Arauca (columns). The dark-red color indicates higher relative amount, while the light-yellow color indicates lower relative amount.

Figure 5 shows four heat maps of VOCs with a VIP ≥ 1 and *p* < 0.05 for chocolates made from cocoa beans fermented for 48, 72, 96, and 120 h. VOCs were ordered from the highest VIP (2-pentanol) to the lowest VIP (2-phenethyl acetate). In this figure, we can see compounds, like 2,6-dimethylpyrazine, 2,3,5-trimethylpyrazine, 3,5-dimethyl-2-ethylpyrazine, 2,3,5,6-tetramethylpyrazine, and acetoin, which are associated with the fine-flavor characteristics such as nuttiness, chocolate, and caramel. Additionally, acetaldehyde, 2-pentanol, ethyl acetate, 1-methoxy-2-propylacetate, and propyl acetate are associated with fruity notes, while benzyl alcohol is associated with floral notes. Therefore, their presence was considered fundamental to the aromatic properties of chocolates. By contrast, compounds such as 3-methyl butanoic acid were found to exhibit undesirable characteristics.

Volatile compounds 1-methoxy-2-propyl acetate, ethyl acetate, and 2,3,5-trimethylpyrazine were predominantly found in chocolates made from cocoa beans fermented for 96 h. Pyrazines and acetaldehyde were found predominantly in chocolates of 120 h fermentation. Chocolates from agroclimatic clusters C1, C4, C7, C8, and C9 from 96 h of fermentation showed high amounts for compounds 2,3,5,6-tretramethylpyrazine and 2,3,5-trimethylpyrazine. This observation could be correlated with the results obtained for nuttiness (Figure 3), which was higher in clusters C1, C8, and C9, followed by C7, as these compounds are directly associated with the nuttiness attribute.

The esters 1-methoxy-2-propyl acetate, ethyl acetate, and 2-phenethyl acetate showed the highest amounts in chocolates made with beans fermented for 96 h, as did pentanol. Conversely, at 120 h, the lowest values were observed for the nine agroclimatic clusters, which could correlate with the decrease in the fruitiness attribute observed for most of the agroclimatic clusters (Figure 3).

### 3.4. Change in Amount of Volatile Compounds Associated with Desirable Aroma Descriptors

The analysis is focused on the volatile compounds with VIP ≥ 1 categorized according to their chemical group to observe their evolution throughout the fermentation process across the diverse agroclimatic cacao production clusters.

#### 3.4.1. Pyrazines

The group of pyrazines, which are fundamental to chocolate’s nutty and roasted flavor notes, is considered one of the most relevant volatile compounds in chocolate, and they could be classified as high-quality key aroma markers. In the present study, the total amounts of pyrazines increased during fermentation, as shown in Figure S4. This study aligns with findings from previous studies suggesting that pyrazines form not only during the roasting process but also as a result of microbiological activities. Specifically, tetramethylpyrazine and trimethylpyrazine are synthesized by *Bacillus subtilis* and *Bacillus megaterium* toward the end of fermentation [16,17,27,42]. Notably, the amounts of 2,3,5,6-tetramethylpyrazine and 2,6-dimethylpyrazine exhibited significant variations throughout the fermentation process. Amounts increased between 96 and 120 h of fermentation across most regions, as shown in Figure S4. However, exceptions were observed in agroclimatic regions C2 and C5, where the pattern did not hold.

#### 3.4.2. Esters

Esters, synthesized during the fermentation process, play a pivotal role in shaping the aromatic profile of chocolates, imparting them with characteristic fruity and floral aromas. These compounds are recognized as significant markers of aroma quality [43]. Throughout fermentation, the amounts of esters generally increase, indicative of aroma development, before experiencing a subsequent decrease, a trend corroborated by various studies [10,12,20]. Specifically, the esters 1-methoxy-propyl acetate and 2-phenethyl acetate, pertinent to chocolate batches from cacao production clusters C1, C3, C4, C5, and C7, followed this pattern, as shown in Figure S5. Notably, these esters reached the peak amount across most agroclimatic regions after 96 h of fermentation, only to diminish as fermentation continued, as shown in Figure S5.

Furthermore, the fruity sensory attributes identified in chocolate samples from 96 and 120 h of fermentation have been directly linked to the presence of these esters during those specific fermentation intervals [10,12]. It has been observed that extending fermentation beyond 96 h leads to a decline in ester production, adversely affecting the aroma quality of chocolate and beans, particularly diminishing the intensity of fruity notes.

#### 3.4.3. Aldehyde and Ketones

Aldehydes, as volatile compounds, offer fruity and floral characteristics that enhance sensory experiences. Notably, the production of acetaldehyde and acetoin during the fermentation process, particularly at 96 and 120 h, has been closely associated with the development of fruity and creamy sensory attributes in chocolate, as shown in Figure S6. Acetaldehyde, in particular, has been recognized as a significant predictor of various sensory attributes, distinguishing itself as a critical compound during fermentation stages. Its amount notably increases with processing time, reflecting a trend consistent with other aldehydes such as 2-methylbutanal, 3-methylbutanal, and benzaldehyde, suggesting a broad impact on the aromatic profile of chocolate [16,27].

Ketones, similarly, are integral to the aromatic appeal of chocolate, with acetoin emerging as a key ketone due to its significant influence on aroma. The amount of acetoin has been observed to increase during fermentation, peaking at approximately 96 h. This pattern aligns with findings from previous studies, reinforcing the importance of aldehydes and ketones in defining the aromatic quality of chocolate [12,27].

#### 3.4.4. Alcohols

Isomer A of 2,3-butanediol was consistently found in higher amounts across all agroclimatic cacao production clusters, with its amount peaking between 96 and 120 h of fermentation, as shown in Figure S7. Conversely, the behavior of 2,3-butanediol Isomer B throughout fermentation exhibited more variability. Our research substantiates the fact that chocolates derived from cocoa seeds fermented for 96–120 h possess volatile compounds strongly correlated with high-quality aroma descriptors, leading to enhanced scores for desirable sensory attributes and overall quality. The observed decline in fine-flavor sensory attributes outside this fermentation window may be attributed to variations in the quantities and types of volatile precursors formed during fermentation, which undergo reactions during the roasting stage, notably through Maillard reactions [27].

Furthermore, the agroclimatic region C9 demonstrated high amounts of acetaldehyde, 2,3-butanediol isomers, and pyrazines, contrasted with lower amounts of the esters 1-methoxy-2-propyl acetate and 2-phenethyl acetate. This region, distinguished by its hotter and drier conditions compared to the markedly rainier region C4, exhibits a distinct geographical and climatic profile. Agroclimatic region C4 displayed different volatile compound marker profiles, such as higher amounts of 1-methoxy-2-propyl acetate, 2-phenethyl acetate, and acetoin compared to region C9. Additionally, sensory profiles varied between these regions; at 96 h, the nuttiness was significantly more pronounced in cluster 9, while fruitiness was slightly higher in cluster 4. However, by 120 h of fermentation, a greater reduction in fruitiness was noted for the cluster. These findings highlight the significant influence of climate and geographical location on the volatile and sensory profiles of chocolate.

## 4. Conclusions

This study advances the field of cocoa fermentation science by exploring the interactions between the agroclimatic conditions of cacao production and fermentation processes and their impact on the sensory profile and volatilome of chocolate.

By analyzing cacao samples from nine different agroclimatic clusters and examining the impact of fermentation time, our results revealed a critical fermentation window of 96 h that optimizes the sensory profile of chocolate, enhancing fine cocoa notes. This period is considered crucial for the development of key volatile compounds that significantly contribute to the aroma and flavor quality of cocoa products. However, the agroclimate variation observed between the nine regions had no significant global impact on the fine-quality attributes evaluated in the chocolates. The identification of specific volatile markers associated with premium flavor profiles provides a novel approach for the industry to evaluate and improve the quality of cocoa beans.

The implications of this study extend to practical applications for cocoa producers and chocolate manufacturers, offering a scientific basis for optimizing fermentation protocols to achieve desired flavor characteristics. In light of these findings, this study advocates for interdisciplinary research to investigate the interplay among cocoa genetic diversity, agroclimatic conditions of cacao production, fermentation methodologies, and the integrated analysis of chemical, aromatic, and sensorial characteristics in chocolate. Such initiatives could deepen our understanding of flavor formation science and promote innovation within the cocoa production chain.

## Figures and Tables

**Table 1:** Volatile compounds identified in the 108 dark chocolate samples, along with their associated flavor descriptors and Kovats retention index.

No.	Volatile Compounds	Odor/ Flavor Descriptions	Chemical Group	RT (min)	KI Lit.	KI Cal.
	Fruity					
1	Acetaldehyde	Tart (acidic), fruity (α)	Aldehyde	2.96	702 ± 12	702
2	2-p entanone	Fruity, thinner, acetone (η)	Ketone	7.00	686	690
3	Nonanal	Tallowy, fruity (η)	Aldehyde	30.14	1390	1398
4	Hexanal	Green, woody (σ)	Aldehyde	11.20	798	800
5	Ethyl acetate	Pineapple (η)	Ester	4.55	872	888
6	Methyl acetate	Fruity (σ)	Ester	3.78	813	828
7	Butyl acetate	Fruity banana, sharp (ω)	Ester	10.26	1075	1070
8	Hexyl acetate	Pear - like (μ)	Ester	22.00	1273 ± 7	1279
9	1-m ethoxy -2-propyl acetate	Sweet (υ)	Ester	42.20	1233	1238
10	2-heptanone	Fruity sweet, coconut (η)	Ketone	15.57	1181	1182
11	2,3-b utanediol (isomer A)	Sweet (α)	Alcohol	39.61	1492–1582	1533
12	2,3-b utanediol (isomer B)	Sweet (α)	Alcohol	39.61	1492–1582	1533
13	2,3-b utanediol monoacetate	–	Ester	41.30	1560–1570	1568
14	2-pentanol	Green, fruity, sweet (η)	Alcohol	13.12	1122	1119
15	Limonene	Citrus (η)		17.10	1026	1030
16	2-phenylacetaldehyde	Berry, geranium, honey (α)	Aldehyde	44.90	1634	1640
	Nutty/chocolate					
17	2-m ethylbutanal	Malty and chocolate (γ)	Aldehyde	5.00	910	914
18	2-m ethyl -propanal	Malty and chocolate (β)	Aldehyde	3.60	817	819
19	Benzaldehyde	Nutty, almond (β)	Aldehyde	36.28	1516	1520
20	3-m ethylbutanal	Malty and chocolate (β)	Aldehyde	5.10	912	918
21	2,3,5,6-t etramethylpyrazine	Milk-coffee, roasted, chocolate (δ)	Pyrazine	34.19	1489	1469
22	2,3,5-t rimethylpyrazine	Cocoa, roasted, cooked peanut (δ)	Pyrazine	29.82	1408	1402
23	Methyl pyrazine	Nutty-chocolate (ε)	Pyrazine	21.70	1266 ± 10	1270
24	2,6 -d imethylpyrazine	Vegetal. roasted (η)	Pyrazine	24.55	1300	1370
25	3,5 -dimethyl -2-ethylpyrazine	Earthy roasty (σ)	Pyrazine	34.00	1464	1470
26	4H- pyran -4-one-2-3-dihydro-3,5-dihydroxy-6-methyl	–	Pyrazine	64.187	2271 ± 14	2281
27	2-formylpyrrole	Malty /dark chocolate (ε)	Pyrrole	61.00	2030 ± 14	2041
28	2-a cetyl -pyrrole	Bread, cocoa, hazelnut, licorice, walnut (δ)	Pyrrole	58.84	1985	1973
29	furan-2-phentyl	Sweet rum, caramel and butter (χ)	Furan	20.00	1232 ± 9	1150
	Floral					
30	2-phenylethyl acetate	Dried fruit–like, flowery (γ)	Ester	53.14	1810	1813
31	Benzyl alcohol	Floral, rose, phenolic (φ)	Alcohol	57.00	1872	1870
32	Phenylmethyl butanoate	Sweet, floral, strawberry, (ε)	Ester	57.30	1860 ± 9	1869
33	2-p henylethanol	Flowery (γ)	Alcohol	57.39	1891	1906
	Creamy/buttery					
34	Acetoin	Buttery, sour cream (δ)	Ketone	22.01	1250	1284
35	Butyrolactone	Sweet, caramel-like (δ)	Lactone	42.18	1618	1632
	Undesirable					
36	3-m ethylbutanoic acid	Pungent, sweaty (γ)	Acid	45.70	1676	1666
37	Acetic acid	Sour, astringent, vinegar (γ)	Acid	31.80	1452	1449
38	Ethanol	Alcoholic (η)	Alcohol	5.59	929	932
39	2-furanmethanol	Bitter (α)	Furan	46.80	1661 ± 9	1650
40	Ethylbenzene	Off -flavor (ε)	Hydrocarbon	13.11	857	856
41	Dimethyl sulfide	Unpleasant, cabbage-like (γ)	Organosulfur	3.30	716	754
42	Toluene	Caramel, synthetic (off-flavor) Fuel-like (α)	Hydrocarbon	9.10	1042 ± 11	1025
	Others					
43	Unidentified compound 1	Unspecified	Unspecified	2.70	–	–
44	Unidentified compound 2	Unspecified	Unspecified	28.10	–	–

(α) [16], (γ) [15], (β) [35], (δ) [36], (φ) [10], (η) [37], (σ) [38], (μ) [39], (ω) [32], (ε) www.thegoodscentscompany.com, accessed on 5 February 2024. (χ) [40], (υ) [41].

## Data Availability

The original contributions presented in the study are included in the article/Supplementary Materials, further inquiries can be directed to the corresponding author.

## Supplementary Materials

The following supporting information can be downloaded at: https://www.mdpi.com/article/10.3390/foods13162614/s1, Figure S1: Sensory profile of chocolates made by fermented cocoa at 48, 72, 96 and 120 h of mixtures of cocoa materials from nine climatic zones of production (C1, C2, C3, C4, C5, C6, C7, C8, and C9); Figure S2: Dynamics of sensory attributes during cocoa fermentation from nine climatic zones of production (C1, C2, C3, C4, C5, C6, C7, C8, and C9) grouped by fermentation time of 48, 72, 96 and 120 h; Figure S3: Permutation test for the dataset [n = 100; R2 = (0.0, 0.133); Q2 = (0.0, −0.715)]; Table S1: Average relative amount (mean ± SD) of volatile compound (µg/g chocolate 1-butanol equivalents) identified in chocolates from samples at 48, 72, 96, and 120 h of fermentation from cocoa beans of the nine climatic zones of production (C1, C2, C3, C4, C5, C6, C7, C8, and C9); Table S2: The highest discriminant compounds and VIP value; Figure S4: Relative amount of the pyrazine significant OAV > 1 and VIP > 1 with significant variations between clusters; Figure S5: Relative amount of the ester significant OAV > 1 and VIP > 1 with significant variations between clusters; Figure S6: Relative amount of the acetaldehyde and acetoine with OAV > 1 and VIP > 1 with significant variations between regions; Figure S7: Relative amount of the alcohol significant VIP > 1 with significant variations between clusters.
