# Supplementary material for: Exploring the Impact of Fermentation Time and Climate on Quality of Cocoa Bean-Derived Chocolate: Sensorial Profile and Volatilome Analysis
Source: Foods. 2024 Aug 20;13(16):2614. doi: 10.3390/foods13162614 (PMC11353615; doi:10.3390/foods13162614)
Supplement: Supplementary file 1 [file foods-13-02614-s001.zip › foods-3071619-supplementary.pdf]

Supplementary Materials

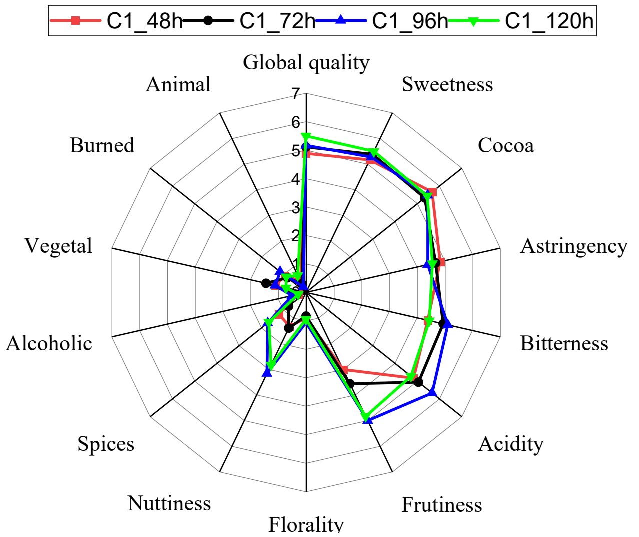

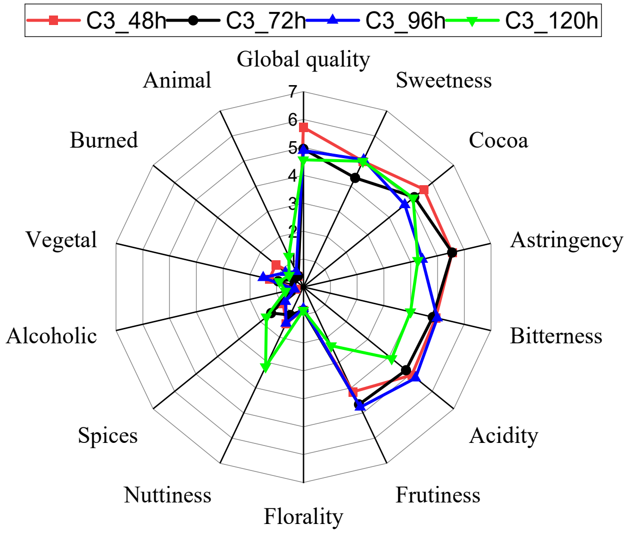

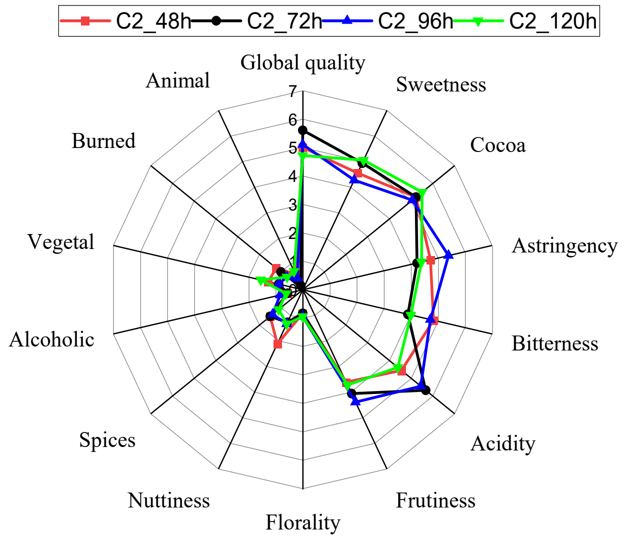

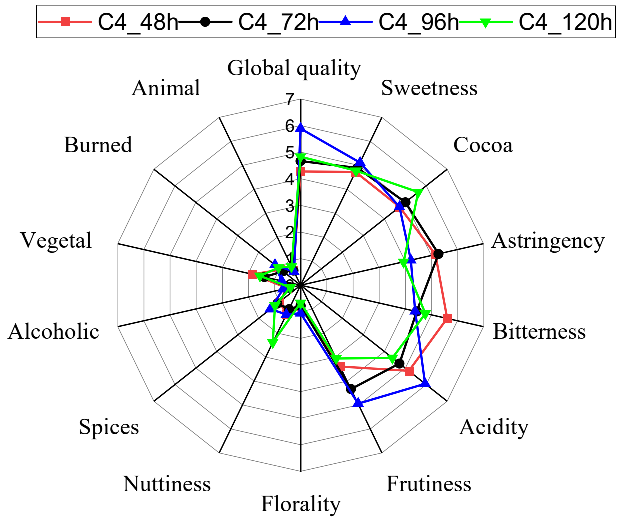

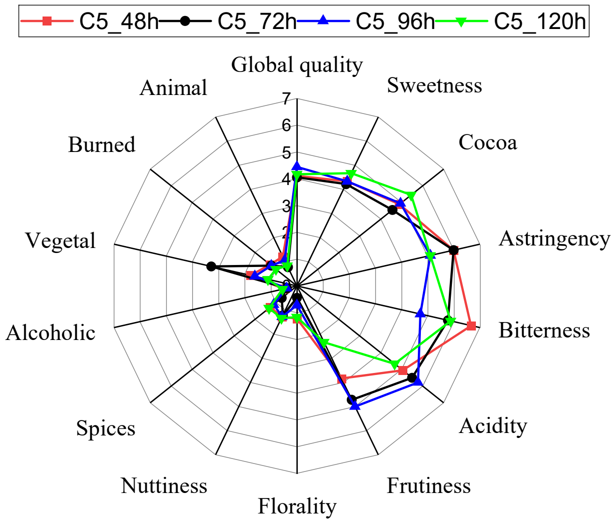

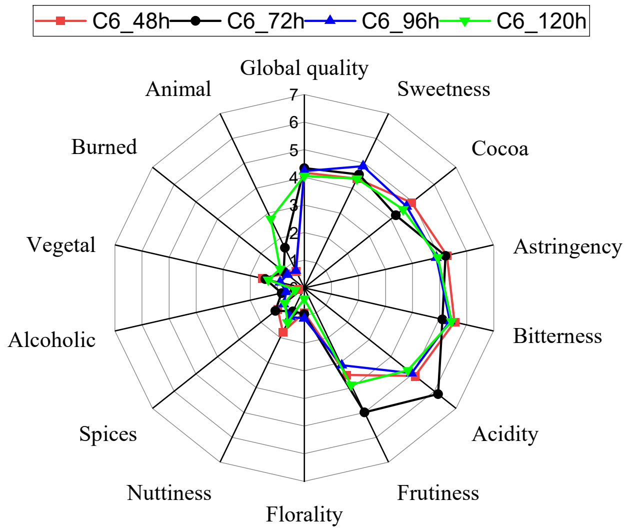

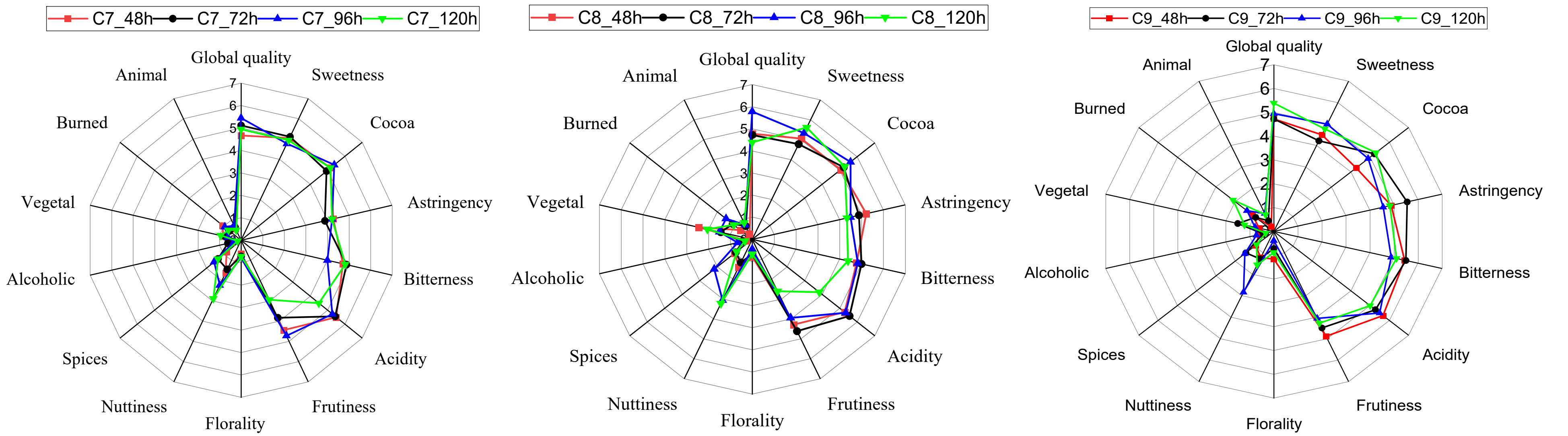

Figure S1. Sensory profile of chocolates made by fermented cocoa at 48, 72, 96 and 120 h o f mixtures of cocoa materials from nine climatic zones of production (C1, C2, C3, C4, C5, C6, C7, C8, and C9).

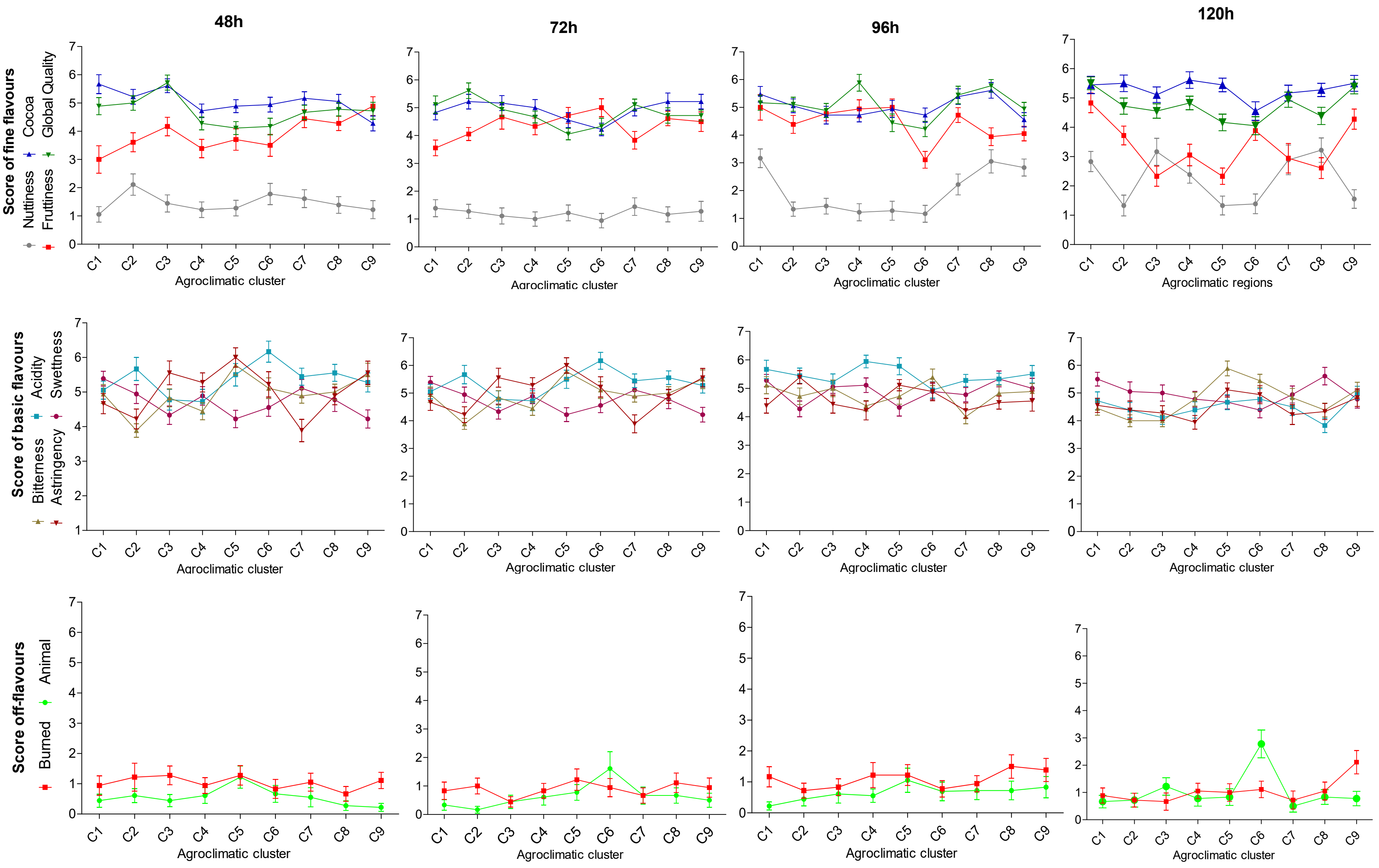

Figure S2. Dynamics of sensory attributes during cocoa fermentation from nine climatic zones of production (C1, C2, C3, C4, C5, C6, C7, C8, and C9) grouped by fermentation time of 48, 72, 96 and 120 hours.

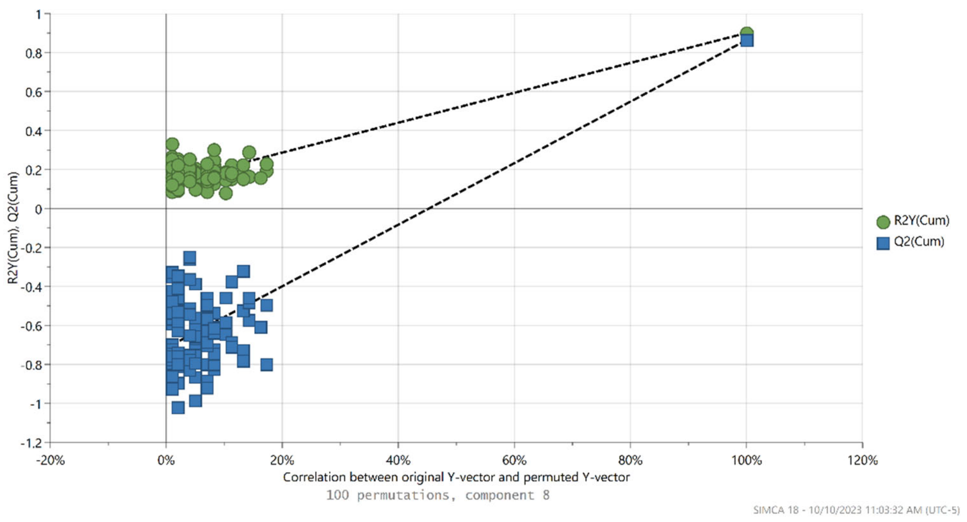

Figure S3. Permutation test for the dataset [n=100; R2 = (0.0, 0.133); Q2 = (0.0, −0.715)].

Table S1. Average concentrations (mean ± SD) of volatile compound (μg/g chocolate 1-butanol equivalents) identified in chocolates from samples at 48, 72, 96, and 120 h of fermentation from cocoa beans of the nine climatic zones of production (C1, C2, C3, C4, C5, C6, C7, C8, and C9).

	48h								
	**C1**	**C2**	**C3**	**C4**	**C5**	**C6**	**C7**	**C8**	**C9**
D-limoneno	0.034 ± 0.005	0.002 ± 0.003	0.004 ± 0.002	0.050 ± 0.002	0.034 ± 0.015	0.013 ± 0.004	0.009 ± 0.002	0.001 ± 0.000	0.002 ± 0.000
Acetaldehyde	0.124 ± 0.023	0.082 ± 0.009	0.080 ± 0.007	0.094 ± 0.005	0.093 ± 0.001	0.098 ± 0.010	0.102 ± 0.009	0.118 ± 0.003	0.111 ± 0.009
2-methyl-propanal	0.487 ± 0.049	0.706 ± 0.034	0.446 ± 0.011	0.379 ± 0.021	0.417 ± 0.044	0.588 ± 0.071	0.467 ± 0.026	0.721 ± 0.035	0.334 ± 0.014
2-methylbutanal	0.945 ± 0.121	1.321 ± 0.050	0.733 ± 0.020	0.731 ± 0.009	1.055 ± 0.014	1.386 ± 0.092	0.939 ± 0.018	1.702 ± 0.015	1.064 ± 0.048
3-methylbutanal	1.860 ± 0.617	3.099 ± 0.014	1.999 ± 0.048	0.831 ± 0.034	0.892 ± 0.197	1.259 ± 0.202	1.337 ± 0.046	2.191 ± 0.158	0.613 ± 0.034
Hexanal	0.280 ± 0.043	0.239 ± 0.005	0.258 ± 0.015	0.116 ± 0.004	0.146 ± 0.006	0.157 ± 0.019	0.220 ± 0.006	0.301 ± 0.026	0.069 ± 0.007
Nonanal	0.280 ± 0.031	0.262 ± 0.032	0.198 ± 0.015	0.167 ± 0.015	0.172 ± 0.025	0.502 ± 0.052	0.328 ± 0.006	0.579 ± 0.022	0.428 ± 0.022
Benzaldehyde	2.671 ± 0.353	1.716 ± 0.226	1.283 ± 0.030	1.201 ± 0.044	1.654 ± 0.029	1.824 ± 0.156	2.402 ± 0.058	2.517 ± 0.069	1.997 ± 0.054
2-phenyl-acetaldehyde	3.849 ± 0.252	4.410 ± 0.601	3.190 ± 0.183	0.974 ± 0.035	1.117 ± 0.056	1.267 ± 0.053	1.532 ± 0.066	1.727 ± 0.157	1.085 ± 0.052
Ethanol	0.396 ± 0.013	5.594 ± 0.041	48.017 ± 0.502	1.299 ± 0.071	4.133 ± 0.045	41.973 ± 0.859	0.924 ± 0.018	3.525 ± 0.077	20.465 ± 0.388
2-pentanol	0.448 ± 0.012	0.509 ± 0.023	0.318 ± 0.013	1.196 ± 0.060	1.520 ± 0.069	0.196 ± 0.017	0.593 ± 0.008	0.823 ± 0.017	0.405 ± 0.017
2-3-butanediol Isomer A	0.632 ± 0.184	1.486 ± 0.168	4.222 ± 0.651	11.159 ± 1.470	0.200 ± 0.006	66.016 ± 1.763	0.167 ± 0.011	0.099 ± 0.009	0.203 ± 0.001
2-3-butanediol Isomer B	20.190 ± 0.246	3.506 ± 0.145	4.546 ± 0.586	5.353 ± 0.028	3.395 ± 0.072	1.609 ± 0.130	3.568 ± 0.276	11.379 ± 0.283	18.694 ± 0.586
2-furanmethanol	0.189 ± 0.005	0.426 ± 0.005	0.464 ± 0.012	0.530 ± 0.009	0.340 ± 0.022	0.671 ± 0.017	0.837 ± 0.058	0.539 ± 0.013	0.582 ± 0.026
Benzyl alcohol	0.148 ± 0.008	0.062 ± 0.005	0.100 ± 0.006	0.096 ± 0.005	0.194 ± 0.002	0.083 ± 0.007	0.083 ± 0.007	0.114 ± 0.015	0.086 ± 0.009
2-phenylethanol	4.139 ± 0.265	0.762 ± 0.046	1.143 ± 0.070	2.161 ± 0.123	2.560 ± 0.370	2.314 ± 0.084	2.258 ± 0.204	1.692 ± 0.179	1.730 ± 0.054
Acetic acid	279.361 ±11.923	112.974 ± 9.199	104.311 ± 9.654	243.653 ± 2.254	193.069 ± 3.355	161.709 ± 2.146	161.122 ± 9.987	191.143 ± 2.282	280.644 ± 3.236
3-methylbutanoic acid	4.688 ± 0.139	2.297 ± 0.052	2.218 ± 0.115	5.335 ± 0.119	2.190 ± 0.056	2.407 ± 0.461	5.177 ± 0.208	1.452 ± 0.102	1.924 ± 0.193
2-pentanone	0.077 ± 0.011	0.323 ± 0.014	0.129 ± 0.011	0.266 ± 0.018	0.780 ± 0.054	0.061 ± 0.005	0.071 ± 0.006	0.146 ± 0.006	0.267 ± 0.019
2-heptanone	0.632 ± 0.017	0.572 ± 0.028	0.167 ± 0.006	0.800 ± 0.072	1.280 ± 0.099	0.840 ± 0.054	0.499 ± 0.014	0.445 ± 0.054	0.698 ± 0.023
Acetoine	48.876 ± 1.766	33.882 ± 0.534	37.531 ± 1.117	60.401 ± 0.701	36.728 ± 1.503	42.244 ± 0.260	19.762 ± 0.701	10.124 ± 0.431	16.813 ± 1.811
Methyl acetate	76.069 ± 1.282	7.268 ± 0.478	3.145 ± 0.013	11.578 ± 0.316	2.827 ± 0.322	5.190 ± 0.081	2.843 ± 0.063	4.658 ± 0.471	5.542 ± 0.125

Ethyl acetate	0.874 ± 0.009	0.769 ± 0.019	1.649 ± 0.031	5.265 ± 0.106	1.464 ± 0.068	0.806 ± 0.038	2.239 ± 0.042	0.0351 ± 0.009	0.269 ± 0.015
Butyl acetate	4.535 ± 0.034	2.578 ± 0.149	0.640 ± 0.011	1.123 ± 0.088	0.406 ± 0.056	0.346 ± 0.022	0.234 ± 0.005	0.687 ± 0.054	0.788 ± 0.125
Hexyl acetate	0.016 ± 0.001	0.070 ± 0.004	0.070 ± 0.004	0.059 ± 0.007	0.057 ± 0.006	0.062 ± 0.003	0.093 ± 0.007	0.130 ± 0.013	0.121 ± 0.001
2,3-butandiol monoacetate	22.158 ± 1.169	6.773 ± 0.161	4.321 ± 0.400	18.753 ± 1.740	4.394 ± 0.132	23.335 ± 1.648	3.672 ± 0.285	6.649 ± 1.344	11.566 ± 0.947
1-methoxy-2-propylacetate	33.575 ± 1.465	5.184 ± 0.112	6.287 ± 0.603	7.576 ± 0.405	6.062 ± 0.174	2.517 ± 0.147	6.711 ± 0.447	20.350 ± 0.616	33.482 ± 0.765
2-phenylethyl acetate	0.945 ± 0.054	0.173 ± 0.019	0.293 ± 0.022	0.352 ± 0.032	0.564 ± 0.069	0.269 ± 0.028	0.419 ± 0.053	0.625 ± 0.031	0.551 ± 0.108
phenylmethyl butanoate	0.041 ± 0.002	0.035 ± 0.002	0.038 ± 0.002	0.041 ± 0.002	0.033 ± 0.006	0.061 ± 0.006	0.022 ± 0.004	0.011 ± 0.001	0.016 ± 0.003
Toluene	0.345 ± 0.037	0.409 ± 0.047	0.390 ± 0.039	0.455 ± 0.032	0.821 ± 0.221	0.919 ± 0.098	0.663 ± 0.056	0.844 ± 0.151	1.464 ± 0.074
Ethylbenzene	0.216 ± 0.009	0.044 ± 0.001	0.056 ± 0.005	0.032 ± 0.003	0.157 ± 0.011	0.432 ± 0.029	0.029 ± 0.004	0.114 ± 0.008	0.303 ± 0.019
Methyl pyrazine	0.201 ± 0.008	0.220 ± 0.017	0.180 ± 0.016	0.193 ± 0.019	0.129 ± 0.015	0.388 ± 0.004	0.317 ± 0.026	0.188 ± 0.021	0.204 ± 0.009
2-6-dimethylpyrazine	0.210 ± 0.010	0.460 ± 0.015	0.300 ± 0.022	0.371 ± 0.005	0.175 ± 0.023	0.593 ± 0.048	0.593 ± 0.053	0.385 ± 0.016	0.323 ± 0.014
2-3-5-trimethylpyhrazine	5.902 ± 0.215	3.330 ± 0.136	1.808 ± 0.258	3.472 ± 0.085	3.314 ± 0.159	4.587 ± 0.597	3.034 ± 0.300	1.477 ± 0.135	2.124 ± 0.248
3-5-dimethyl-2-ethylpyrazine	0.205 ± 0.007	0.243 ± 0.030	0.151 ± 0.013	0.507 ± 0.072	0.314 ± 0.019	0.500 ± 0.013	0.224 ± 0.025	0.162 ± 0.033	0.212 ± 0.003
2-3-5-6-tetramethylpyrazine	37.861 ± 2.119	5.424 ± 0.154	8.725 ± 0.952	20.767 ± 0.666	9.325 ± 0.410	28.808 ± 0.964	3.352 ± 0.339	1.109 ± 0.056	2.575 ± 0.091
4H-pyran-4-one- 2-3-dihydro-3-5-dihydroxy-6-methyl	0.759 ± 0.016	0.937 ± 0.067	1.285 ± 0.022	0.909 ± 0.104	0.724 ± 0.091	1.049 ± 0.006	1.277 ± 0.155	1.420 ± 0.107	1.750 ± 0.178
2-acetyl-pyrrol	1.600 ± 0.567	1.700 ± 0.235	1.040 ± 0.279	1.223 ± 0.120	1.358 ± 0.608	1.219 ± 0.427	1.503 ± 0.442	2.271 ± 0.097	0.705 ± 0.272
2-formylpyrrole	0.038 ± 0.004	0.049 ± 0.024	0.029 ± 0.015	0.044 ± 0.021	0.036 ± 0.008	0.026 ± 0.001	0.040 ± 0.011	0.053 ± 0.008	0.026 ± 0.004
furan-2-penthyl	0.293 ± 0.022	0.287 ± 0.110	0.312 ± 0.016	0.202 ± 0.089	0.293 ± 0.075	0.307 ± 0.038	0.166 ± 0.020	0.288 ± 0.141	0.227 ± 0.014
Dimethyl sulfide	0.156 ± 0.042	0.406 ± 0.025	0.241 ± 0.096	0.188 ± 0.077	0.290 ± 0.117	0.514 ± 0.019	0.457 ± 0.221	0.454 ± 0.070	0.306 ± 0.080
Unidentified compound 1	1.406 ± 0.326	1.644 ± 0.407	1.084 ± 0.345	1.366 ± 0.356	1.741 ± 0.251	1.345 ± 0.122	1.386 ± 0.270	1.438 ± 0.065	1.315 ± 0.097
Unidentified compound 2	0.083 ± 0.066	0.040 ± 0.046	0.010 ± 0.016	0.548 ± 0.374	0.071 ± 0.006	0.094 ± 0.025	0.043 ± 0.054	0.002 ± 0.001	0.076 ± 0.062
Butyrolactone	0.934 ± 0.102	0.625 ± 0.033	0.927 ± 0.412	0.377 ± 0.038	0.539 ± 0.155	0.433 ± 0.021	1.062 ± 0.653	0.537 ± 0.019	0.345 ± 0.058

	72h								
	**C1**	**C2**	**C3**	**C4**	**C5**	**C6**	**C7**	**C8**	**C9**
D-limoneno	0.035 ± 0.005	0.002 ± 0.000	0.034 ± 0.006	0.059 ± 0.004	0.037 ± 0.014	0.074 ± 0.004	0.053 ± 0.007	0.031 ± 0.004	0.020 ± 0.003
Acetaldehyde	0.181 ± 0.003	0.053 ± 0.002	0.084 ± 0.010	0.073 ± 0.001	0.079 ± 0.005	0.147 ± 0.004	0.117 ± 0.009	0.100 ± 0.008	0.073 ± 0.005
2-methyl-propanal	0.689 ± 0.036	0.783 ± 0.053	0.490 ± 0.046	0.506 ± 0.013	0.495 ± 0.028	0.669 ± 0.041	0.504 ± 0.026	0.778 ± 0.036	0.630 ± 0.034
2-methylbutanal	1.275 ± 0.011	1.853 ± 0.030	0.790 ± 0.147	1.363 ± 0.001	1.318 ± 0.065	1.488 ± 0.005	1.115 ± 0.015	1.837 ± 0.018	1.407 ± 0.106
3-methylbutanal	2.420 ± 0.121	1.094 ± 0.042	2.007 ± 0.308	0.640 ± 0.025	0.884 ± 0.118	1.417 ± 0.144	0.899 ± 0.048	1.747 ± 0.068	1.658 ± 0.120
Hexanal	0.243 ± 0.005	0.042 ± 0.005	0.150 ± 0.006	0.042 ± 0.008	0.164 ± 0.044	0.113 ± 0.003	0.082 ± 0.006	0.123 ± 0.002	0.197 ± 0.070
Nonanal	0.440 ± 0.005	0.312 ± 0.025	0.164 ± 0.025	0.295 ± 0.025	0.187 ± 0.010	0.292 ± 0.006	0.321 ± 0.035	0.217 ± 0.033	0.663 ± 0.054
Benzaldehyde	2.721 ± 0.121	1.594 ± 0.063	1.322 ± 0.246	1.797 ± 0.067	1.953 ± 0.031	2.016 ± 0.044	3.126 ± 0.271	2.642 ± 0.197	2.340 ± 0.075
2-phenyl-acetaldehyde	3.390 ± 0.188	1.406 ± 0.017	3.136 ± 0.508	1.128 ± 0.100	1.080 ± 0.064	1.344 ± 0.068	1.373 ± 0.241	2.188 ± 0.187	2.204 ± 0.345
Ethanol	141.495 ± 0.417	33.477 ± 1.486	46.195 ± 0.794	12.240 ± 0.038	1.195 ± 0.010	14.463 ± 0.705	0.791 ± 0.014	9.481 ± 0.397	1.112 ± 0.033
2-pentanol	1.725 ± 0.038	0.868 ± 0.025	0.802 ± 0.009	1.751 ± 0.022	3.415 ± 0.021	2.169 1.081	1.131 ± 0.061	0.734 ± 0.029	1.778 ± 0.104
2-3-butanediol Isomer A	0.256 ± 0.035	0.350 ± 0.144	0.250 ± 0.297	24.405 ± 0.868	0.141 ± 0.026	0.259 ± 0.007	0.336 ± 0.020	0.307 ± 0.020	0.251 ± 0.016
2-3-butanediol Isomer B	5.357 ± 0.519	1.327 ± 0.302	2.776 ± 0.341	10.540 ± 0.329	8.164 ± 1.045	16.563 ± 0.011	11.565 ± 0.478	7.006 ± 0.096	1.987 ± 0.077
2-furanmethanol	0.270 ± 0.017	0.918 ± 0.082	0.175 ± 0.018	0.179 ± 0.004	0.196 ± 0.009	0.228 ± 0.001	0.748 ± 0.051	0.530 ± 0.009	0.480 ± 0.014
Benzyl alcohol	0.128 ± 0.003	0.070 ± 0.014	0.069 ± 0.004	0.085 ± 0.000	0.107 ± 0.007	0.133 ± 0.011	0.116 ± 0.006	0.124 ± 0.010	0.151 ± 0.021
2-phenylethanol	3.449 ± 0.290	0.671 ± 0.106	1.216 ± 0.066	2.004 ± 0.009	2.291 ± 0.324	1.931 ± 0.061	3.084 ± 0.175	2.472 ± 0.180	0.662 ± 0.041
Acetic acid	385.637 ± 11.92	207.262 ±2.199	217.622 ± 6.654	233.399± 16.961	213.906 ±18.576	337.649± 0.292	344.276 ± 6.866	185.393 ± 7.947	192.941 ± 6.091
3-methylbutanoic acid	8.329 ± 0.118	8.274 ± 0.520	8.044 ± 0.287	4.706 ± 0.386	2.494 ± 0.347	2.210 ± 0.070	4.999 ± 0.107	9.807 ± 0.850	8.718 ± 0.067
2-pentanone	0.255 ± 0.016	0.085 ± 0.006	0.051 ± 0.002	0.160 ± 0.003	0.584 ± 0.048	1.640 ± 0.013	0.201 ± 0.008	0.204 ± 0.006	0.077 ± 0.003
2-heptanone	0.796 ± 0.039	0.439 ± 0.036	0.216 ± 0.023	0.561 ± 0.097	1.656 ± 0.109	1.543 ± 0.060	0.700 ± 0.018	0.877 ± 0.011	0.396 ± 0.004
Acetoine	56.825 ± 1.329	19.113 ± 1.591	26.950 ± 1.855	34.427 ± 0.728	46.916 ± 4.039	46.922 ± 0.850	42.298 ± 1.083	42.012 ± 2.559	40.585 ± 0.653
Methyl acetate	86.331 ± 1.695	1.242 ± 0.089	2.090 ± 0.061	11.429 ± 0.405	11.737 ± 1.084	8.847 2.784	9.683 ± 0.196	10.589 ± 1.310	14.722 ± 1.304
Ethyl acetate	12.535 ± 2.705	1.585 ± 0.088	5.839 ± 0.302	8.221 ± 0.127	7.707 ± 0.143	27.228 ± 0.368	5.511 ± 0.091	2.771 ± 0.150	0.269 ± 0.021
Butyl acetate	7.580 ± 0.485	0.083 ± 0.006	0.135 ± 0.013	0.904 ± 0.097	0.960 ± 0.305	1.720 ± 0.610	0.424 ± 0.012	0.974 ± 0.216	2.351 ± 0.054
Hexyl acetate	0.029 ± 0.004	0.121 ± 0.006	0.024 ± 0.002	0.016 ± 0.004	0.025 ± 0.005	0.027 ± 0.011	0.077 ± 0.008	0.116 ± 0.005	0.079 ± 0.001
2,3-butandiol monoacetate	8.694 ± 0.579	2.650 ± 0.481	3.652 ± 0.302	18.741 ± 0.468	10.848 ± 1.157	16.855 ± 0.540	14.294 ± 0.603	5.674 ± 0.506	3.048 ± 0.528
1-methoxy-2-propylacetate	8.916 ± 0.517	1.768 ± 0.266	4.244 ± 0.348	16.671 ± 0.353	14.625 ± 2.140	29.437 ± 0.491	22.061 ± 0.645	9.663 ± 0.179	3.742 ± 0.128
2-phenylethyl acetate	0.584 ± 0.096	0.115 ± 0.017	0.179 ± 0.005	0.590 ± 0.010	0.519 ± 0.063	0.569 ± 0.136	0.921 ± 0.095	0.586 ± 0.043	0.169 ± 0.008

phenylmethyl butanoate	0.054 ± 0.006	0.025 ± 0.003	0.023 ± 0.001	0.031 ± 0.003	0.019 ± 0.004	0.019 ± 0.000	0.031 ± 0.003	0.015 ± 0.006	0.016 ± 0.002
Toluene	0.395 ± 0.044	0.952 ± 0.110	0.512 ± 0.056	0.171 ± 0.024	0.599 ± 0.111	0.765 ± 0.016	0.471 ± 0.088	1.145 ± 0.107	0.563 ± 0.056
Ethylbenzene	0.099 ± 0.005	0.050 ± 0.006	0.054 ± 0.000	0.024 ± 0.002	0.105 ± 0.003	0.398 ± 0.011	0.023 ± 0.004	0.128 ± 0.017	0.013 ± 0.003
Methyl pyrazine	0.203 ± 0.022	0.199 ± 0.040	0.099 ± 0.015	0.080 ± 0.011	0.064 ± 0.017	0.076 ± 0.004	0.240 ± 0.003	0.314 ± 0.004	0.189 ± 0.007
2-6-dimethylpyrazine	0.288 ± 0.013	0.439 ± 0.068	0.082 ± 0.006	0.085 ± 0.003	0.088 ± 0.023	0.089 ± 0.013	0.343 ± 0.010	0.422 ± 0.028	0.423 ± 0.010
2-3-5-trimethylpyhrazine	6.927 ± 0.439	4.856. ± 0.250	1.213 ± 0.055	2.219 ± 0.066	2.802 ± 0.012	3.654 ± 0.624	6.096 ± 0.222	5.474 ± 0.304	4.345 ± 0.217
3-5-dimethyl-2-ethylpyrazine	0.602 ± 0.043	0.149 ± 0.021	0.099 ± 0.005	0.473 ± 0.043	0.380 ± 0.048	0.466 ± 0.076	0.505 ± 0.019	0.275 ± 0.061	0.328 ± 0.058
2-3-5-6-tetramethylpyrazine	19.588 ± 2.144	3.052 ± 0.446	4.958 ± 0.199	15.839 ± 0.560	20.291 ± 3.385	28.076 1.884	27.676 ± 1.277	17.636 ± 0.264	8.494 ± 0.635
4H-pyran-4-one- 2-3-dihydro-3-5-dihydroxy-6-methyl	1.204 ± 0.142	1.258 ± 0.310	0.494 ± 0.048	0.577 ± 0.028	0.651 ± 0.063	0.788 ± 0.130	1.088 ± 0.167	1.437 ± 0.160	1.025 ± 0.192
2-acetyl-pyrrol	1.343 ± 0.521	1.088 ± 0.329	1.090 ± 0.265	0.478 ± 0.199	1.091 ± 0.204	1.203 ± 0.425	1.196 ± 0.369	0.891 ± 0.086	1.891 ± 0.414
2-formylpyrrole	0.035 ± 0.003	0.025 ± 0.002	0.044 ± 0.017	0.041 ± 0.007	0.034 ± 0.011	0.045 ± 0.000	0.037 ± 0.014	0.057 ± 0.015	0.040 ± 0.009
furan-2-penthyl	0.412 ± 0.029	0.449 ± 0.030	0.328 ± 0.102	0.154 ± 0.013	0.365 ± 0.018	0.140 ± 0.097	0.183 ± 0.036	0.415 ± 0.011	0.368 ± 0.046
Dimethyl sulfide	0.844 ± 0.217	0.641 ± 0.214	0.835 ± 0.294	0.338 ± 0.060	0.642 ± 0.405	0.182 ± 0.046	0.129 ± 0.003	0.357 ± 0.208	0.137 ± 0.031
Unidentified compound 1	1.328 ± 0.097	1.193 ± 0.090	1.374 ± 0.048	1.062 ± 0.215	1.424 ± 0.083	1.385 ± 0.007	1.218 ± 0.172	1.171 ± 0.108	1.593 ± 0.264
Unidentified compound 2	0.135 ± 0.026	0.105 ± 0.055	0.085 ± 0.017	0.173 ± 0.094	0.351 ± 0.562	0.277 ± 0.245	0.268 ± 0.148	0.307 ± 0.005	0.068 ± 0.008
Butyrolactone	0.995 ± 0.083	0.820 ± 0.105	0.808 ± 0.163	0.366 ± 0.292	0.597 ± 0.076	0.523 ± 0.009	0.437 ± 0.073	0.348 ± 0.024	1.143 ± 0.140

	**96h**								
	C1	C2	C3	C4	C5	C6	C7	C8	C9
D-limoneno	0.057 ± 0.004	0.023 ± 0.002	0.013 ± 0.008	0.068 ± 0.002	0.088 ± 0.011	0.036 ± 0.010	0.076 ± 0.007	0.034 ± 0.003	0.024 ± 0.006
Acetaldehyde	0.101 ± 0.003	0.083 ± 0.008	0.100 ± 0.058	0.115 ± 0.008	0.069 ± 0.006	0.061 ± 0.001	0.088 ± 0.005	0.065 ± 0.006	0.066 ± 0.002
2-methyl-propanal	0.628 ± 0.019	0.438 ± 0.017	0.667 ± 0.031	0.519 ± 0.020	0.702 ± 0.091	0.667 ± 0.019	0.614 ± 0.024	0.665 ± 0.020	0.713 ± 0.134
2-methylbutanal	1.097 ± 0.021	0.804 ± 0.022	1.447 ± 0.025	0.943 ± 0.002	1.327 ± 0.171	1.656 ± 0.081	1.102 ± 0.009	1.542 ± 0.046	1.884 ± 0.107
3-methylbutanal	2.159 ± 0.084	1.566 ± 0.057	1.249 ± 0.064	1.675 ± 0.083	2.262 ± 0.030	1.053 ± 0.091	1.371 ± 0.030	1.209 ± 0.082	1.307 ± 0.226
Hexanal	0.179 ± 0.003	0.101 ± 0.006	0.057 ± 0.008	0.149 ± 0.006	0.203 ± 0.006	0.070 ± 0.002	0.228 ± 0.007	0.193 ± 0.062	0.226 ± 0.083
Nonanal	0.164 ± 0.004	0.302 ± 0.010	0.207 ± 0.009	0.271 ± 0.018	0.210 ± 0.008	0.368 ± 0.011	0.447 ± 0.008	0.359 ± 0.082	0.345 ± 0.015
Benzaldehyde	1.619 ± 0.024	1.293 ± 0.060	1.372 ± 0.036	1.687 ± 0.038	2.107 ± 0.036	1.988 ± 0.115	2.427 ± 0.179	1.752 ± 0.090	2.179 ± 0.030

2-phenyl-acetaldehyde	2.636 ± 0.040	2.221 ± 0.088	1.371 ± 0.006	1.582 ± 0.113	2.726 ± 0.216	1.467 ± 0.304	1.803 ± 0.124	1.379 ± 0.073	1.866 ± 0.154
Ethanol	127.005 ± 2.239	6.013 ± 0.374	36.569 ± 3.658	10.622 ± 0.152	4.291 ± 0.213	4.559 ± 0.982	0.662 ± 0.012	0.609 ± 0.041	0.400 ± 0.013
2-pentanol	0.603 ± 0.049	0.524 ± 0.024	0.569 ± 0.020	0.321 ± 0.005	1.656 ± 0.138	1.916 ± 1.657	1.213 ± 0.023	0.375 ± 0.007	0.356 ± 0.008
2-3-butanediol Isomer A	0.253 ± 0.052	88.348 ±11.644	16.312 ± 0.287	12.700 ± 1.868	0.232 ± 0.012	0.305 ± 0.097	0.475 ± 0.023	0.236 ± 0.068	0.250 ± 0.033
2-3-butanediol Isomer B	6.3192 ± 0.522	6.034 ± 0.423	6.080 ± 0.786	18.636 ± 0.399	11.120 ± 0.456	1.511 ± 0.433	6.455 ± 0.338	1.471 ± 0.327	2.177 ± 0.157
2-furanmethanol	0.389 ± 0.011	0.188 ± 0.004	0.221 ± 0.044	0.398 ± 0.034	0.386 ± 0.021	0.375 ± 0.143	0.931 ± 0.013	0.795 ± 0.057	0.531 ± 0.025
Benzyl alcohol	0.171 ± 0.015	0.109 ± 0.010	0.061 ± 0.009	0.140 ± 0.005	0.099 ± 0.006	0.115 ± 0.018	0.152 ± 0.017	0.103 ± 0.013	0.124 ± 0.003
2-phenylethanol	3.328 ± 0.331	0.624 ± 0.042	0.908 ± 0.156	1.892 ± 0.073	2.058 ± 0.261	1.834 ± 0.122	3.473 ± 0.430	0.963 ± 0.058	0.818 ± 0.068
Acetic acid	343.399 ± 8.429	195.489 ± 6.674	153.435 ± 3.116	315.956 ± 1.270	194.099 ± 7.329	178.488 ± 7.942	565.533 ± 22.01[illegible]	210.364 ± 3.131	280.643 ± 3.369
3-methylbutanoic acid	14.358 ± 1.425	9.762 ± 0.166	7.725 ± 0.158	6.046 ± 0.455	9.403 ± 0.696	5.965 ± 3.095	4.921 ± 0.227	9.496 ± 1.712	9.410 ± 0.943
2-pentanone	0.110 ± 0.015	0.083 ± 0.007	0.028 ± 0.004	0.037 ± 0.005	0.350 ± 0.028	0.714 ± 0.632	0.210 ± 0.004	0.071 ± 0.002	0.115 ± 0.010
2-heptanone	0.645 ± 0.035	0.285 ± 0.003	0.122 ± 0.024	0.407 ± 0.013	1.426 ± 0.095	1.211 ± 0.061	0.588 ± 0.009	0.404 ± 0.025	0.395 ± 0.041
Acetoine	49.111 ± 1.587	37.086 ± 0.597	37.145 ± 0.210	41.191 ± 0.544	57.473 ± 3.832	58.313 ±10.847	61.130 ± 2.735	39.788 ± 0.851	39.600 ±3.877
Methyl acetate	19.884 ± 1.688	3.307 ± 0.128	2.519 ± 0.521	9.834 ± 0.492	10.744 ± 1.094	9.157 ± 1.705	14.344 ± 0.188	17.218 ± 0.276	4.439 ± 0.867
Ethyl acetate	19.991 ± 1.053	2.068 ± 0.063	9.552 ± 0.235	1.506 ± 0.111	19.937 ± 1.454	21.999 ± 2.348	2.180 ± 0.032	9.424 ± 0.078	14.429 ± 1.208
Butyl acetate	0.773 ± 0.093	0.284 ± 0.034	0.142 ± 0.022	0.527 ± 0.051	1.404 ± 0.130	1.231 ± 0.813	0.447 ± 0.340	1.450 ± 0.056	0.468 ± 0.114
Hexyl acetate	0.038 ± 0.001	0.020 ± 0.001	0.026 ± 0.005	0.046 ± 0.007	0.082 ± 0.008	0.100 ± 0.016	0.083 ± 0.001	0.100 ± 0.009	0.094 ± 0.007
2,3-butandiol monoacetate	14.579 ± 0.946	24.862 ± 0.564	6.566 ± 1.479	15.061 ± 0.781	5.295 ± 1.331	10.991 ± 3.210	17.060 ± 2.100	4.738 ± 0.665	5.567 ± 0.149
1-methoxy-2-propylacetate	19.306 ± 1.125	4.873 ± 0.142	4.013 ± 0.082	17.162 ± 0.922	7.680 ± 0.440	14.291 ± 1.898	31.823 ± 2.426	3.644 ± 0.119	2.619 ± 0.136
2-phenylethyl acetate	1.207 ± 0.096	0.158 ± 0.022	0.220 ± 0.034	0.763 ± 0.060	0.651 ± 0.057	0.434 ± 0.088	1.371 ± 0.185	0.206 ± 0.050	0.243 ± 0.015
phenylmethyl butanoate	0.048 ± 0.002	0.025 ± 0.003	0.021 ± 0.005	0.036 ± 0.005	0.020 ± 0.001	0.027 ± 0.007	0.044 ± 0.011	0.029 ± 0.003	0.025 ± 0.005
Toluene	0.287 ± 0.060	0.408 ± 0.035	0.786 ± 0.114	0.254 ± 0.038	0.881 ± 0.285	0.813 ± 0.239	0.531 ± 0.039	0.820 ± 0.007	1.241 ± 0.156
Ethylbenzene	0.192 ± 0.004	0.099 ± 0.001	0.083 ± 0.008	0.125 ± 0.014	0.188 ± 0.005	0.150 ± 0.157	0.032 ± 0.000	0.032 ± 0.006	0.065 ± 0.004
Methyl pyrazine	0.148 ± 0.004	0.116 ± 0.014	0.131 ± 0.010	0.123 ± 0.001	0.119 ± 0.020	0.111 ± 0.027	0.282 ± 0.011	0.475 ± 0.016	0.185 ± 0.014
2-6-dimethylpyrazine	0.195 ± 0.009	0.106 ± 0.005	0.100 ± 0.016	0.138 ± 0.003	0.188 ± 0.019	0.168 ± 0.062	0.512 ± 0.032	0.821 ± 0.063	0.437 ± 0.054
2-3-5-trimethylpyhrazine	5.338 ± 0.350	5.067 ± 0.265	3.730 ± 0.158	7.318 ± 0.295	3.334 ± 0.198	8.80 ± 0.451	9.686 ± 0.347	6.112 ± 0.597	15.349 ± 0.309
3-5-dimethyl-2-ethylpyrazine	0.485 ± 0.048	0.182 ± 0.009	0.327 ± 0.029	0.737 ± 0.038	0.408 ± 0.075	0.517 ± 0.057	0.898 ± 0.038	0.499 ± 0.156	0.407 ± 0.060

2-3-5-6-tetramethylpyrazine	33.995 ± 2.623	11.194 ± 0.266	9.574 ± 1.090	20.858 ± 2.012	14.791 ± 2.065	28.414 ± 2.717	42.268 ± 3.530	11.432 ± 1.726	41.144 ± 1.182
4H-pyran-4-one- 2-3-dihydro-3-5-dihydroxy-6-methyl	1.356 ± 0.044	0.581 ± 0.075	0.850 ± 0.032	1.145 ± 0.033	1.489 ± 0.169	0.888 ± 0.236	1.667 ± 0.436	1.527 ± 0.091	1.448 ± 0.217
2-acetyl-pyrrol	1.604 ± 0.389	1.605 ± 0.145	1.943 ± 0.342	1.578 ± 0.085	2.020 ± 0.359	1.494 ± 0.823	1.559 ± 0.163	1.566 ± 0.132	2.716 ± 0.258
2-formylpyrrole	0.059 ± 0.016	0.048 ± 0.013	0.041 ± 0.005	0.044 ± 0.003	0.048 ± 0.010	0.038 ± 0.004	0.038 ± 0.007	0.042 ± 0.002	0.069 ± 0.015
furan-2-penthyl	0.221 ± 0.025	0.357 ± 0.115	0.450 ± 0.100	0.403 ± 0.132	0.149 ± 0.053	0.113 ± 0.057	0.167 ± 0.005	0.250 ± 0.007	0.116 ± 0.007
Dimethyl sulfide	0.144 ± 0.007	0.350 ± 0.210	0.353 ± 0.053	0.360 ± 0.178	0.227 ± 0.082	0.165 ± 0.044	0.129 ± 0.003	0.169 ± 0.008	0.098 ± 0.008
Unidentified compound 1	1.344 ± 0.134	1.532 ± 0.278	1.359 ± 0.130	1.226 ± 0.084	1.383 ± 0.033	1.526 ± 0.179	1.377 ± 0.085	1.295 ± 0.061	1.758 ± 0.092
Unidentified compound 2	0.199 ± 0.194	0.500 ± 0.592	0.361 ± 0.389	0.272 ± 0.161	0.234 ± 0.188	0.082 ± 0.051	0.126 ± 0.005	0.154 ± 0.006	0.126 ± 0.020
Butyrolactone	1.147 ± 0.091	0.987 ± 0.152	1.023 ± 0.256	1.135 ± 0.139	1.044 ± 0.240	1.101 ± 0.602	0.355 ± 0.040	0.456 ± 0.010	0.651 ± 0.029

	**120h**								
	C1	C2	C3	C4	C5	C6	C7	C8	C9
D-limoneno	0.005 ± 0.001	0.003 ± 0.001	0.002 ± 0.000	0.052 ± 0.001	0.028 ± 0.012	0.024 ± 0.017	0.054 ± 0.002	0.022 ± 0.011	0.018 ± 0.003
Acetaldehyde	0.120 ± 0.007	0.093 ± 0.003	0.140 ± 0.007	0.305 ± 0.040	0.207 ± 0.015	0.137 ± 0.007	0.235 ± 0.015	0.250 ± 0.060	0.331 ± 0.036
2-methyl-propanal	0.592 ± 0.023	0.456 ± 0.066	0.606 ± 0.023	0.488 ± 0.029	0.420 ± 0.026	0.594 ± 0.023	0.496 ± 0.057	0.656 ± 0.174	0.494 ± 0.046
2-methylbutanal	1.204 ± 0.028	0.867 ± 0.024	1.149 ± 0.040	0.949 ± 0.018	0.715 ± 0.010	1.187 ± 0.265	1.116 ± 0.095	1.075 ± 0.234	0.998 ± 0.019
3-methylbutanal	1.472 ± 0.041	1.233 ± 0.036	1.424 ± 0.095	1.463 ± 0.067	1.215 ± 0.065	1.428 ± 0.409	1.351 ± 0.684	1.650 ± 0.327	0.532 ± 0.025
Hexanal	0.157 ± 0.006	0.107 ± 0.004	0.139 ± 0.004	0.136 ± 0.004	0.324 ± 0.013	0.157 ± 0.015	0.274 ± 0.090	0.253 ± 0.038	0.042 ± 0.004
Nonanal	0.252 ± 0.017	0.209 ± 0.016	0.313 ± 0.022	0.263 ± 0.036	0.265 ± 0.026	0.283 ± 0.047	0.177 ± 0.023	0.556 ± 0.166	0.345 ± 0.016
Benzaldehyde	3.081 ± 0.106	1.976 ± 0.209	2.512 ± 0.103	2.432 ± 0.107	2.077 ± 0.062	2.483 ± 0.477	2.502 ± 0.072	2.153 ± 0.104	3.221 ± 0.055
2-phenyl-acetaldehyde	2.364 ± 0.219	1.718 ± 0.049	2.399 ± 0.035	2.259± 0.114	1.903 ± 0.044	1.718 ± 0.172	1.560± 0.182	2.944 ± 0.675	1.364 ± 0.111
Ethanol	45.677 ± 2.213	72.051 ± 11.050	67.127 ± 2.788	7.123 ± 0.309	24.478 ± 0.179	0.378 ± 0.168	7.977 ± 0.233	1.466 ± 0.197	49.122 ± 2.018
2-pentanol	0.174 ± 0.009	0.131 ± 0.003	0.061 ± 0.001	0.185 ± 0.002	0.116 ± 0.037	0.495 ± 0.328	0.159 ± 0.006	0.078 ± 0.022	0.087 ± 0.013
2-3-butanediol Isomer A	11.987 ± 2.050	163.036 ±12.474	110.894 ± 8.056	43.556± 4.398	60.841 ± 15.002	12.398 ± 4.188	1.629 ± 0.319	88.817± 16.698	120.193 ±10.358
2-3-butanediol Isomer B	1.712 ± 0.119	1.712 ± 0.128	0.971 ± 0.243	2.085± 0.188	1.555 ± 0.312	1.902 ± 0.085	1.586 ± 0.124	1.309 ± 0.310	1.689 ± 0.140
2-furanmethanol	0.394 ± 0.010	0.177 ± 0.018	0.422 ± 0.023	0.506 ± 0.010	0.473 ± 0.020	0.658 ± 0.172	0.251 ± 0.027	0.858 ± 0.205	0.862 ± 0.009
Benzyl alcohol	0.173 ± 0.015	0.116 ± 0.011	0.164 ± 0.012	0.182 ± 0.011	0.136 ± 0.014	0.155 ± 0.006	0.203 ± 0.021	0.125 ± 0.008	0.222 ± 0.006
2-phenylethanol	2.142 ± 0.175	1.737 ± 0.180	1.614 ± 0.076	1.999 ± 0.127	2.093 ± 0.139	1.932 ± 0.053	2.436 ± 0.223	1.183 ± 0.083	2.061 ± 0.053

Acetic acid	149.010 ± 1.283	122.851 ± 7.851	95.124 ± 3.730	169.595 ± 3.459	95.933 ± 0.964	177.191 ± 18.286	70.434 ± 0.759	126.652 ±23.342	184.765 ± 4.896
3-methylbutanoic acid	8.751 ± 0.347	16.168 ± 0.088	15.453 ± 0.309	15.483± 1.548	13.100 ± 0.206	11.226 ± 3.099	0.082 ± 0.141	14.375 ± 3.569	19.955 ± 0.232
2-pentanone	0.057 ± 0.002	0.040 ± 0.008	0.050 ± 0.006	0.081 ± 0.002	0.056 ± 0.006	0.166 ± 0.181	0.109 ± 0.029	0.040 ± 0.001	0.039 ± 0.003
2-heptanone	0.316 ± 0.010	0.183 ± 0.031	0.114 ± 0.013	0.296 ± 0.012	1.138 ± 0.056	0.834 ± 0.510	0.461 ± 0.045	0.267 ± 0.059	0.232 ± 0.009
Acetoine	11.225 ± 0.111	18.094 ± 0.357	13.414 ± 0.467	46.437 ± 1.278	18.460 ± 0.119	36.847 ± 4.132	24.720 ± 3.392	18.110 ± 0.617	31.000 ± 1.116
Methyl acetate	12.281 ± 0.345	3.487 ± 0.329	3.309 ± 0.190	5.766 ± 0.398	1.237 ± 0.103	5.725 ± 2.481	10.112 ± 1.990	4.321 ± 1.008	5.274 ± 0.170
Ethyl acetate	3.738 ± 0.092	1.048 ± 0.021	0.435 ± 0.050	0.522 ± 0.028	0.102 ± 0.021	0.352 ± 0.285	0.621 ± 0.513	0.129 ± 0.024	0.877 ± 0.029
Butyl acetate	1.640 ± 0.077	0.865 ± 0.043	0.286 ± 0.022	0.330 ± 0.034	0.050 ± 0.008	0.370 ± 0.294	0.641 ± 0.628	0.169 ± 0.029	0.457 ± 0.031
Hexyl acetate	0.075 ± 0.003	0.027 ± 0.002	0.060 ± 0.002	0.068 ± 0.009	0.037 ± 0.001	0.098 ± 0.019	0.045 ± 0.006	0.104 ± 0.023	0.091 ± 0.005
2,3-butandiol monoacetate	12.729 ± 0.497	11.102 ± 1.076	19.779 ± 1.750	17.746± 1.098	12.209 ± 0.501	16.654 ± 7.782	1.393 ± 0.406	13.378 ± 3.461	20.429 ± 1.123
1-methoxy-2-propylacetate	2.680 ± 0.145	1.813 ± 0.102	1.171 ± 0.105	4.289 ± 0.196	1.294 ± 0.020	4.483 ± 2.347	1.438 ± 0.186	1.741 ± 0.008	1.996 ± 0.072
2-phenylethyl acetate	0.283 ± 0.040	0.366 ± 0.028	0.185 ± 0.013	0.377 ± 0.019	0.129 ± 0.013	0.495 ± 0.035	0.294 ± 0.037	0.156 ± 0.011	0.298 ± 0.031
phenylmethyl butanoate	0.049 ± 0.003	0.046 ± 0.004	0.057 ± 0.007	0.047± 0.003	0.040 ± 0.006	0.046 ± 0.016	0.050 ± 0.008	0.033± 0.004	0.056± 0.004
Toluene	0.459 ± 0.014	0.556 ± 0.072	1.162 ± 0.068	0.514 ± 0.004	0.521 ± 0.030	0.856 ± 0.079	0.939 ± 0.070	0.589 ± 0.162	0.706 ± 0.042
Ethylbenzene	0.082 ± 0.004	0.094 ± 0.011	0.221 ± 0.006	0.094 ± 0.005	0.188 ± 0.022	0.033 ± 0.039	0.214 ± 0.034	0.021 ± 0.006	0.111 ± 0.021
Methyl pyrazine	0.545 ± 0.024	0.430 ± 0.005	0.512 ± 0.070	0.275 ± 0.008	0.313 ± 0.007	0.298 ± 0.110	0.360 ± 0.040	0.540 ± 0.109	0.589 ± 0.027
2-6-dimethylpyrazine	1.002 ± 0.035	0.430 ± 0.005	0.695 ± 0.034	0.482 ± 0.019	0.327 ± 0.006	0.593 ± 0.371	0.386 ± 0.083	0.605 ± 0.080	1.299 ± 0.019
2-3-5-trimethylpyhrazine	3.491 ± 0.196	3.717 ± 0.168	3.884 ± 0.026	7.118 ± 0.465	4.686 ± 0.041	5.231 ± 0.452	6.407 ± 1.713	7.235 ± 0.920	4.477 ± 0.680
3-5-dimethyl-2-ethylpyrazine	0.406 ± 0.051	0.404 ± 0.014	0.340 ± 0.006	1.341 ± 0.041	0.348 ± 0.010	0.724 ± 0.038	0.784 ± 0.224	0.461 ± 0.151	0.498 ± 0.137
2-3-5-6-tetramethylpyrazine	2.926 ± 0.384	9.701 ± 1.012	4.986 ± 0.404	21.629 ± 0.547	22.395 ± 0.456	16.898 ± 9.094	29.501 ± 6.558	27.568 ± 2.962	11.302 ± 0.743
4H-pyran-4-one- 2-3-dihydro-3-5-dihydroxy-6-methyl	1.164 ± 0.086	0.831 ± 0.085	1.096 ± 0.166	1.389± 0.049	0.427 ± 0.086	1.364 ± 0.314	0.727 ± 0.088	0.762 ± 0.053	1.322 ± 0.137
2-acetyl-pyrrol	0.845 ± 0.043	1.678 ± 0.192	1.092 ± 0.227	0.666 ± 0.088	0.911 ± 0.101	0.753 ± 0.026	0.517 ± 0.113	1.229 ± 0.314	1.037 ± 0.145
2-formylpyrrole	0.060 ± 0.021	0.044 ± 0.006	0.040 ± 0.023	0.034 ± 0.009	0.060 ± 0.013	0.043 ± 0.008	0.026 ± 0.003	0.074 ± 0.011	0.046 ± 0.007
furan-2-penthyl	0.240 ± 0.090	0.293 ± 0.019	0.074 ± 0.037	0.076 ± 0.004	0.166 ± 0.120	0.119 ± 0.003	0.124 ± 0.052	0.174 ± 0.065	0.065 ± 0.032
Dimethyl sulfide	0.457 ± 0.107	0.161 ± 0.009	0.117 ± 0.017	0.254 ± 0.019	0.283 ± 0.189	0.452 ± 0.140	0.284 ± 0.067	0.350 ± 0.121	0.146 ± 0.014

Unidentified compound 1	2.160 ± 0.592	1.628 ± 0.082	1.086 ± 0.055	1.245± 0.028	1.590 ± 0.929	1.264 ± 0.111	1.080 ± 0.170	1.654 ± 0.088	1.277 ± 0.153
Unidentified compound 2	0.089 ± 0.065	0.149 ± 0.007	0.072 ± 0.024	0.220± 0.040	0.158 ± 0.094	0.148 ± 0.013	0.092 ± 0.019	0.109 ± 0.005	0.098 ± 0.113
Butyrolactone	0.395 ± 0.089	0.465 ± 0.027	0.357 ± 0.039	0.248 ± 0.022	0.350 ± 0.083	0.272 ± 0.007	0.399 ± 0.024	0.564 ± 0.240	0.530 ± 0.116

Table S2. The highest discriminant compounds and VIP value.

Volatile Compound	VIP
2-phenethyl-acetate	1.05
2,6-dimethylpyrazine	1.07
3-methylbutanoic acid	1.12
2,3 butanediol (Isomer B)	1.15
3,5-dimethyl-2-ethylpyrazine	1.2
Acetaldehyde	1.22
Acetic acid	1.23
D-limonene	1.23
2,3 butanediol (Isomer A)	1.24
1-methoxy-2-propyl acetate	1.24
Ethyl acetate	1.25
2,3,5-trimethylpyrazine	1.26
Benzyl alcohol	1.27
2,3,5,6-tetramethylpyrazine	1.33
Acetoin	1.46
2-pentanol	1.48

Change in concentration of volatile compounds associated with desirable aroma descriptors

Pyrazines

2,6-dimethylpyrazine showed higher concentrations for clusters C9, C7, C1 and C8 and 2,3,5,6-tetramethylpyrazine showed higher concentrations for C7, C1 and C6 in chocolate samples from 96 hours of fermentation. Compounds 2,3,5,6-tetramethylpyrazine and 2,3,5-trimethylpyrazine were predominantly found in chocolates at 96 hours, exhibiting similar behavior across nine agroclimatic regions as we can see in Figure S4.

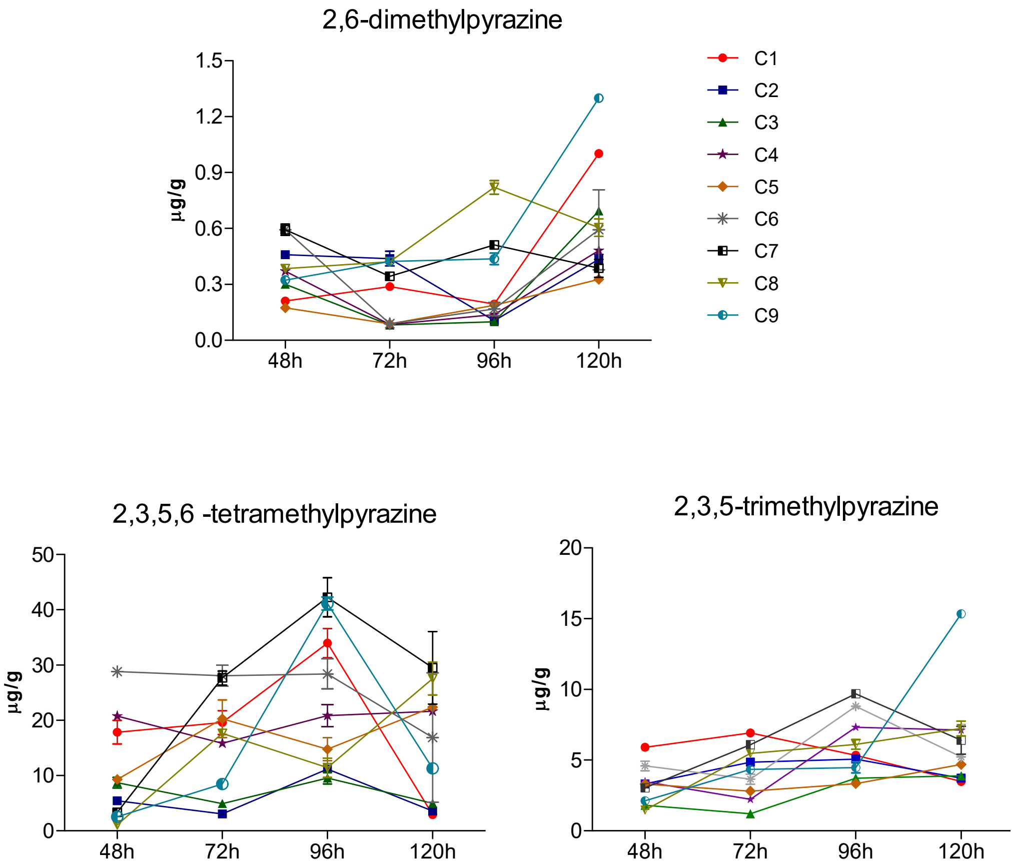

Figure S4. Concentrations of the pyrazine significant OAV>1 and VIP >1 with significant variations between clusters.

For a fermentation time of 120 hours, 2,3,4-trimethylpyrazine exhibited a higher concentration in cluster 9.

Esters

Ester concentrations significantly increased as fermentation time progressed (Ho et al., 2018), followed mostly by a decrease, as also was reported previously. This pattern was observed for the 1-methoxy-propylacetate and 2-penethyl-acetate ester. These esters were found in higher concentrations in agroclimatic regions C1, C4, C5, C7, and C3 after 96 hours of fermentation (Figure S5).

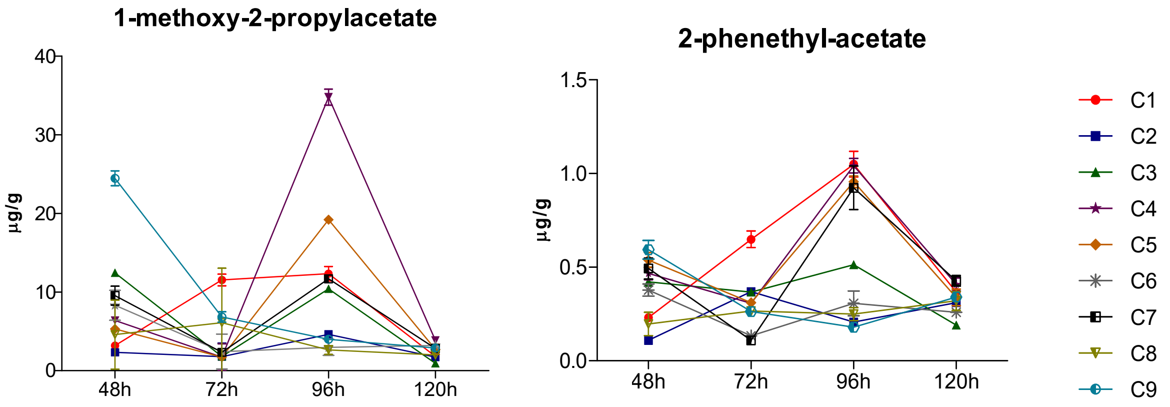

Figure S5. Concentrations of the ester significant OAV>1 and VIP >1 with significant variations between clusters.

Aldehyde and ketones

Acetaldehyde was found in higher concentrations in agroclimatic regions C5, C7, C8, C4, and C9, at a fermentation time of 120 hours, where the maximum concentration was observed-(Figure S6).

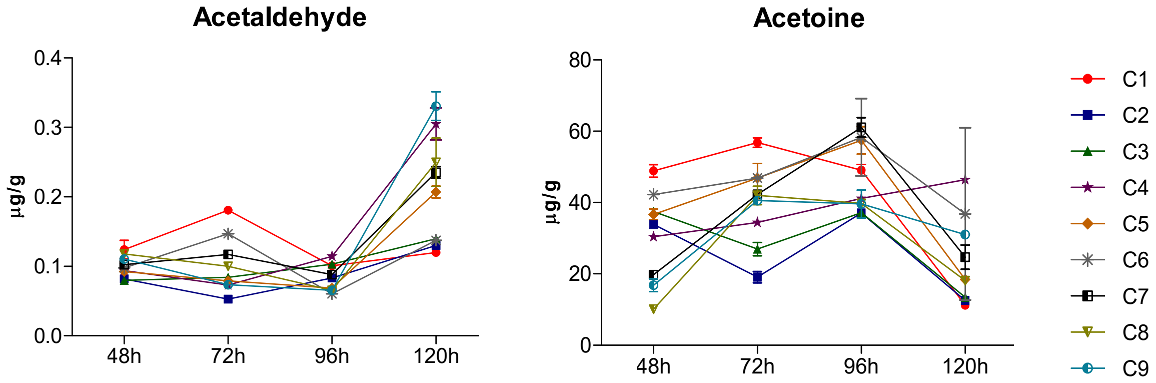

Figure S6. Concentrations of the acetaldehyde and acetoine with OAV>1 and VIP >1 with significant variations between regions.

Acetoin concentration increases with fermentation up to 96 h. This result was similar to those previously reported by (Assi-Clair et al., 2019; Santander et al., 2021).

Alcohols

This trend was observed in all regions except in regions C1, C6, and C7, where the increase was minimal and it was found at elevated levels in cluster C2, as shown in Figure S7.

The 2,3-butanediol isomer B showed a more variable behaviour through fermentation. Increases were observed during the fermentation process for clusters C1, C4, C5, and C7 between 72 and 96 hours of fermentation. For the other clusters, no increases were observed in the concentration between 48 and 120 hours of fermentation. Cluster C9 presented a particular behaviour, as it showed a decrease in the concentration of the isomer B.

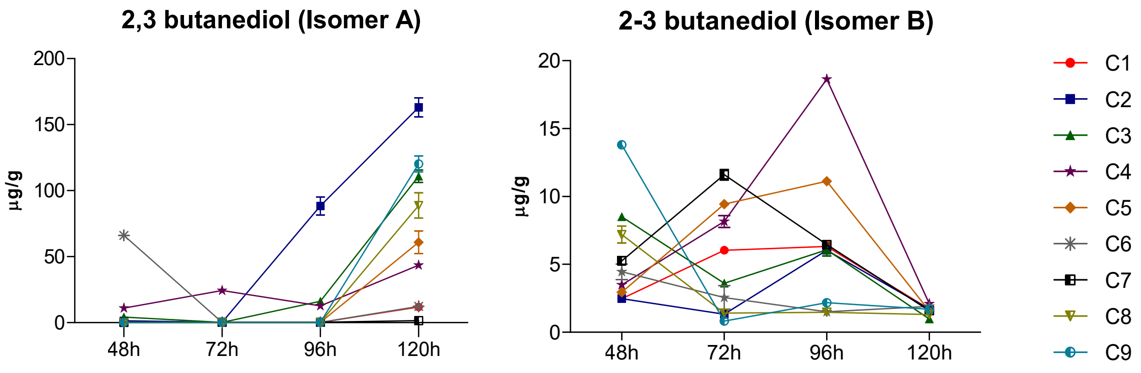

Figure S7. Concentrations of the alcohol significant VIP >1 with significant variations between clusters.
